# Pyrene-Fused Poly-Aromatic Regioisomers: Synthesis, Columnar Mesomorphism, and Optical Properties

**DOI:** 10.3390/molecules28041721

**Published:** 2023-02-10

**Authors:** Qing Zeng, Shuai Liu, Hang Lin, Ke-Xiao Zhao, Xiao-Yan Bai, Ke-Qing Zhao, Ping Hu, Bi-Qin Wang, Bertrand Donnio

**Affiliations:** 1 College of Chemistry and Materials Science, Sichuan Normal University, Chengdu 610066, China; 2 Institut de Physique et Chimie des Matériaux de Strasbourg (IPCMS), CNRS-Université de Strasbourg (UMR 7504), 67034 Strasbourg, France

**Keywords:** polycyclic aromatic hydrocarbons (PAHs), pyrene, columnar mesophase, isomer, fluorescence, homeotropic alignment

## Abstract

π-Extended pyrene compounds possess remarkable luminescent and semiconducting properties and are being intensively investigated as electroluminescent materials for potential uses in organic light-emitting diodes, transistors, and solar cells. Here, the synthesis of two sets of pyrene-containing π-conjugated polyaromatic regioisomers, namely 2,3,10,11,14,15,20,21-octaalkyloxypentabenzo[*a*,*c*,*m*,*o*,*rst*]pentaphene (**BBP***n*) and 2,3,6,7,13,14,17,18-octaalkyloxydibenzo[*j*,*tuv*]phenanthro [9,10-*b*]picene (**DBP***n*), is reported. They were obtained using the Suzuki–Miyaura cross-coupling in tandem with Scholl oxidative cyclodehydrogenation reactions from the easily accessible precursors 1,8- and 1,6-dibromopyrene, respectively. Both sets of compounds, equipped with eight peripheral aliphatic chains, self-assemble into a single hexagonal columnar mesophase, with one short-chain **BBP***n* homolog also exhibiting another columnar mesophase at a lower temperature, with a rectangular symmetry; **BBP***n* isomers also possess wider mesophase ranges and higher mesophases’ stability than their **DBP***n* homologs. These polycyclic aromatic hydrocarbons all show a strong tendency of face-on orientation on the substrate and could be controlled to edge-on alignment through mechanical shearing of interest for their implementation in photoelectronic devices. In addition, both series **BBP***n* and **DBP***n* display green-yellow luminescence, with high fluorescence quantum yields, around 30%. In particular, **BBP***n* exhibit a blue shift phenomenon in both absorption and emission with respect to their **DBP***n* isomers. DFT results were in good agreement with the optical properties and with the stability ranges of the mesophases by confirming the higher divergence from the flatness of **DBP***n* compared with **BBP***n*. Based on these interesting properties, these isomers could be potentially applied not only in the field of fluorescent dyes but also in the field of organic photoelectric semiconductor materials as electron transport materials.

## 1. Introduction

Pyrene and its derivatives are well known for their remarkable photophysical and electronic properties, such as fluorescence with high quantum yields, excimer-based luminescence, piezo-chromic and mechano-chromic fluorescence, and aggregation-induced emission (AIE) behaviors [1,2,3,4,5,6]. π-Extended pyrene compounds with high luminescent and semiconducting properties have been investigated as electroluminescent materials to be applied in organic light-emitting diodes (OLEDs), transistors (OFETs), and solar cells (OSCs) [7,8,9]. Fluorescence and electronic behaviors of molecules strongly depend on their molecular packing and specific interactions with their environment; hence, their association with liquid crystals has been found highly attractive and promising in the perspective of materials science to, for instance, control these interactions and their processing into thin films.

Of particular interest in this field of research are discotic liquid crystals (DLCs), which usually form columnar nanostructures through self-assembly, and as such represent a unique type of soft material with anisotropic one-dimensional electrical and photoconductive properties [10,11,12,13,14,15,16,17,18,19,20,21]. DLCs display higher charge-carrier mobility when compared with that of amorphous silicon, π-conjugated polymers, and polydomain crystalline materials [18], with advantageous features of easier processing into thin-film electronic devices [10]. In the bulk discotic mesomorphic states, electronic charge carriers can efficiently hop along the one-dimensional conductive columns, while the electronic transport between the columns is blocked, as the saturated flexible chains around the rigid discotic cores naturally act as insulators. Thus, the enhanced charge-carrier transport performances of DLCs rely on this antinomic “rigid–flexible” nature of these molecular structures, which are generally composed of a large, central π-conjugated core to which six to eight peripheral alkyl chains are grafted.

Despite the great qualities of pyrene, surprisingly very few pyrene-containing liquid crystals have been reported so far. Due to its natural pseudo-circular shape, pyrene is intrinsically conducive to the formation of discotic systems, but its insertion within calamitic or bent structures has not been detrimental to mesomorphism induction. At first, 1-functionalized pyrene was reported to produce dimeric liquid crystals, symmetrical and non-symmetrical, and to yield essentially nematic (also including the so-called twist–bend nematic) and smectic phases [22,23,24,25]. Pyrene was also inserted in cyclophanes (via 1,6-positions), i.e., pyrenophanes, also yielding a nematic phase [26,27,28], as well as in polycatenars [29] and, recently, in some bent-core mesogens [30], for which columnar mesophases were systematically induced. Dendrimers bearing apical mono- and disubstituted pyrene moiety [31,32,33,34,35,36,37,38,39] have also been reported to self-organize into columnar and cubic mesophases. Lamello-columnar phases were reported for some pyrene with 1,6- and 1,8-disubstitution [40,41,42].

**Chart 1:**
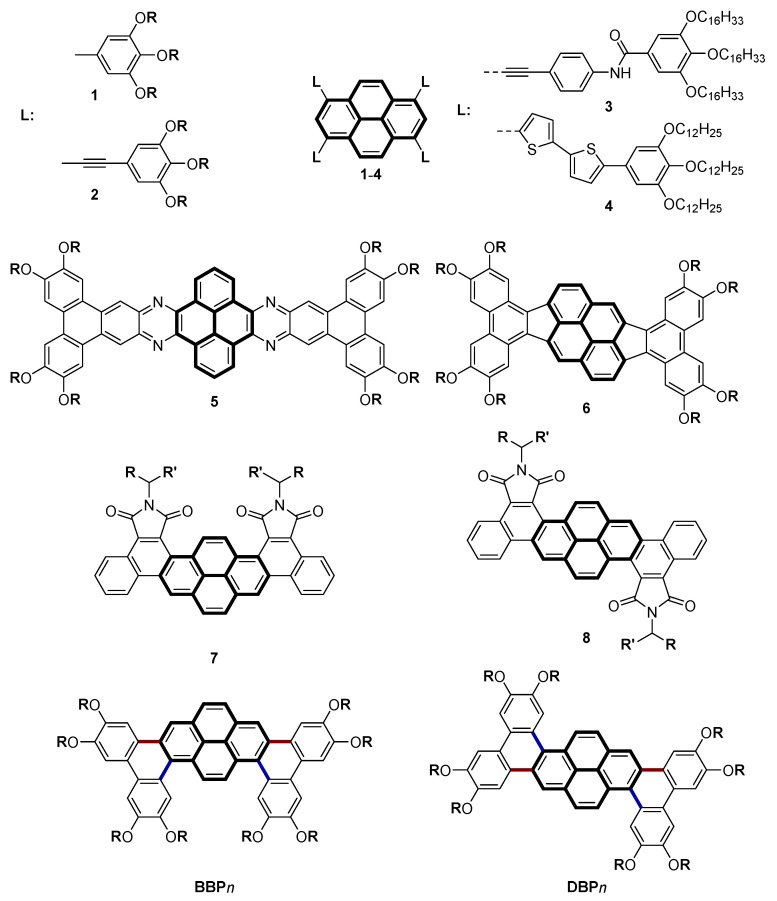
Various examples of pyrene-containing columnar polyaromatic liquid crystals: (**1**) [46,47], (**2**) [45], (**3**) [8], (**4**) [44], (**5**) [58], (**6**) [57], and (**7**,**8**) [60]; **BBP***n* and **DBP***n*, this work (O**R**/O**R′** = OC*_n_*H_2*n*+1,_ *n* = 5–12; NH_2_CH**RR′**, **R** = **R′** = C_5_H_11_, C_11_H_22_ and **R** ≠ **R′** [60]).

DLCs based on pyrene can be designed through the selective multi-substitution of the pyrenic core or via its association with classical discotic cores, as strong promoters for columnar-phase induction. As for the first approach, the most commonly used substitution pattern in the design of pyrene-cored X-/star-shaped mesogens is the 1,3,6,8-tetrasubstitution, essentially because of its facile synthetic accessibility [8,43,44,45,46,47] (**1**–**4**, Chart 1); however, as exceptions, mesogens with 4,5,9,10-tetrasubsititution [48] and 2,4,5,7,9,10-hexasubstitution [49] have also been reported. In the second approach, pyrene (Py) has been reported to combine with “work-horse” discogens, such as hexabenzocoronene (HBC-Py) [50], triphenylene (TP-Py) [51,52], and phthalocyanine (Pc-Py) [53,54], to co-self-assemble into columnar mesophases, and/or with two-dimensional segregated crystalline assemblies of the different discogens on HOPG solid–liquid interfaces, as observed directly using scanning tunneling microscopy (STM) techniques [50,51,52].

Some of these pyrene-based discotic molecules have been found to exhibit unidimensional long-range (time of flight (TOF)-measured) charge-carrier hopping rate (*μ*) ranging from 10^−5^ up to 10^−2^ cm^2^·V^−1^·s^−1^. In addition, some DLC pyrene compounds have shown high fluorescence quantum yields in both solution and solid states [6,8], were found to possess intriguing mechano-chromic luminescent properties [44] and also to behave as supramolecular organogelators [8], fluorescence sensors [1], as well as fluorescent ferroelectric liquid crystals [55,56].

As seen from the various types of structures reviewed above, the current research on pyrene DLCs mainly focuses on methods of functionalizing pyrene by connecting various functional building blocks with single or acetylenic triple bonds to produce X-/star-shaped compounds (e.g., **1**–**4**, Chart 1), while π-conjugated systems fused via the annulation of aromatic rings together (e.g., **5**–**8**, Chart 1) are very limited due to the synthetic methods and reduced solubility of the resulting large DLCs [1,2,3]. Moreover, the positional/regional isomers of polycyclic aromatic hydrocarbons (PAHs) generally exhibit versatile physical properties, such as solid-state π–π stacking modes, electronic properties, mesomorphism, gelation, fluorescence behavior, and photoconductivity/device performance. Such comparative studies are surprisingly not that common (see below); nevertheless, they could be highly relevant in the field of organic electronics.

Herein, we report the successful implementation of the synthesis of two series of liquid crystal isomers based on pyrene equipped with eight long peripheral alkoxy chains, namely **BBP*n*** and **DBP*n***, respectively, through the Suzuki–Miyaura coupling/Scholl oxidative cyclization tandem strategy. Their liquid crystalline behavior and photophysical properties were investigated in great detail, and the structure–property relationship was analyzed using the density functional theory (DFT).

## 2. Results

### 2.1. Molecular Designing, Synthesis, and Characterization

The chemistry of pyrene is mainly based on electrophilic aromatic substitution reactions, and it can be 1-monosubstituted, 1,6-disubstituted, and 1,3,6,8-tetrasubstituted [1,2,3]. Pyrene-cored dendrimers synthesis needs 1-monosubstituted or 1,6-disubstituted pyrene [31,32,33,34,35,36,37,38,39] as starting materials, while X-/star-shaped pyrene derivatives are usually synthesized from 1,3,6,8-tetrabromopyrene (**1**–**4**, Chart 1) [8,43,44,45,46,47]. Recently, Kumar et al. [57] used 1,6-dibromopyrene and disubstituted arylethynylene in Pd-catalyzed coupling reaction in tandem with Scholl cyclopentannulation, to synthesize novel π-extended PAHs (**6**, Chart 1), which exhibit a wide temperature range of columnar mesophases. Eichhorn et al. and Kaafarani et al. [58,59] reported tetraketopyrene condensation with diamino-terphenylene/diamiono-triphenylene for synthesizing board-shaped quinoxalinophenanthrophenazine derivatives (**5**, Chart 1), which were also found to exhibit broad columnar mesophases in addition to strong fluorescence properties. Bock et al. [60] reported isomeric dinaphthopyrene–tetracarboxdiimides (**7** and **8**, Chart 1) that exhibit distinct optical, electrochemical, and mesomorphic properties. They were found to self-assemble into columnar mesophases with hexagonal and rectangular symmetry (depending on the nature of the chains R and R′), the non-centrosymmetric diimides **7** having much larger mesophase ranges than their centrosymmetric counterparts, **8**. Recently, our groups have successfully applied the combination of Suzuki cross-coupling [61] and Scholl reaction [62] in tandem together for the construction of various new π-extended aromatic DLC systems, based on triphenylene [63,64], thiophene and fused thiophene [65,66,67,68], benzothienobenzothiophene (BTBT) [69], carbazole [70], fluorene [70], dibenzothiophene [71], naphthalene [72], and pyridine [73].

**Scheme 1:**
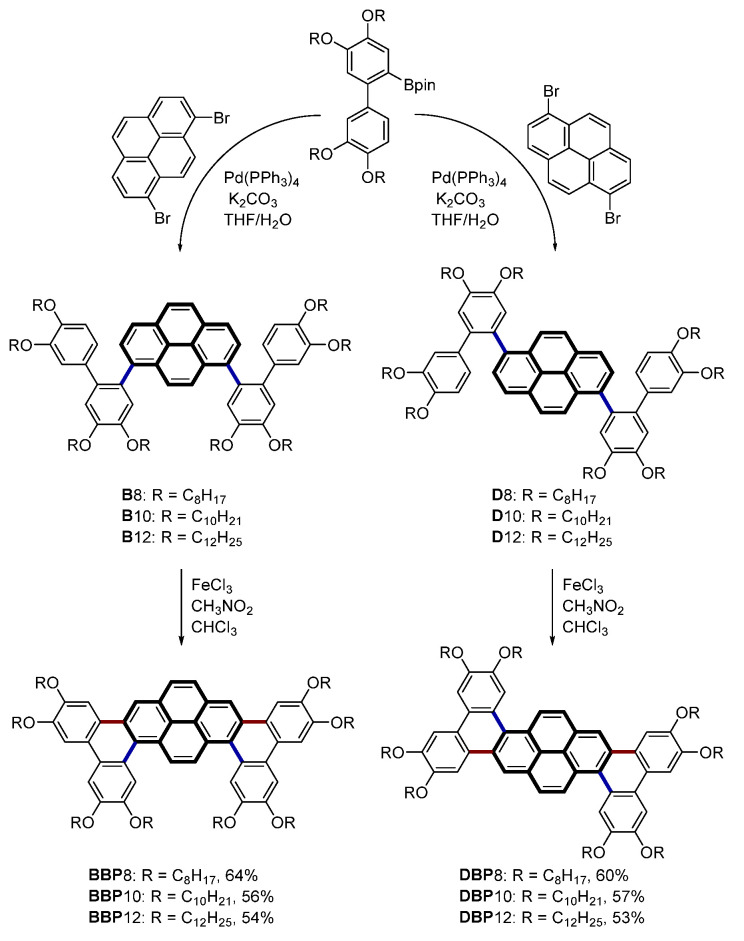
Synthetic route, nomenclature, and overall yields to the pyrene-containing **BBP***n* and **DBP***n* isomeric derivatives.

To further expand the scope of our synthetic strategy adapted to the construction of novel π-extended DLCs, we initiated the synthesis of new pyrene-based PAHs and investigated the impact of topological isomerism on the liquid crystalline and fluorescence properties. This pair of isomeric structures based on pyrene, i.e., **BBP***n* and **DBP***n* (*n* = 8, 10, and 12), was designed from commercial starting materials, i.e., 1,8-dibromopyrene and 1,6-dibromopyrene, respectively, and were obtained in two steps from readily synthesized appropriate precursors (Scheme 1). Firstly, 1,8- and 1,6-dibromopyrene were reacted with readily accessible 4,4,5,5-tetramethyl-2-(3′,4,4′,5-tetrakis(alkoxy)-[1,1′-biphenyl]-2-yl)-1,3,2-dioxaborolane by Pd-catalyzed Suzuki–Miyaura cross-coupling to give non-annulated **B***n* and **D***n* derivatives in good yields, respectively. Subsequently, Scholl oxidative cyclodehydrogenation promoted by FeCl_3_ generated the corresponding fused compounds 2,3,10,11,14,15,20,21-octaalkyloxypentabenzo[*a*,*c*,*m*,*o*,*rst*]pentaphene (**BBP***n*) and 2,3,6,7,13,14,17,18-octaalkyloxydibenzo[*j*,*tuv*]phenanthro[9,10-*b*]picene (**DBP***n*) in ca. 50–60% overall yields on average. Since the molecular symmetry of both isomers is different (*C_2v_* for **BBP***n* and *C_2h_* for **DBP***n*), both sets of compounds are expected to exhibit different physical and chemical properties [60]. All target molecules were fully characterized using ^1^H NMR (Figures S8–S12), ^13^C NMR (Figures S13–S18), elemental analysis, and HRMS (Figures S19–S30).

### 2.2. Liquid Crystalline Properties

#### 2.2.1. Thermal Stability and Mesomorphic Properties by TGA, DSC, and POM

**Figure 1:**
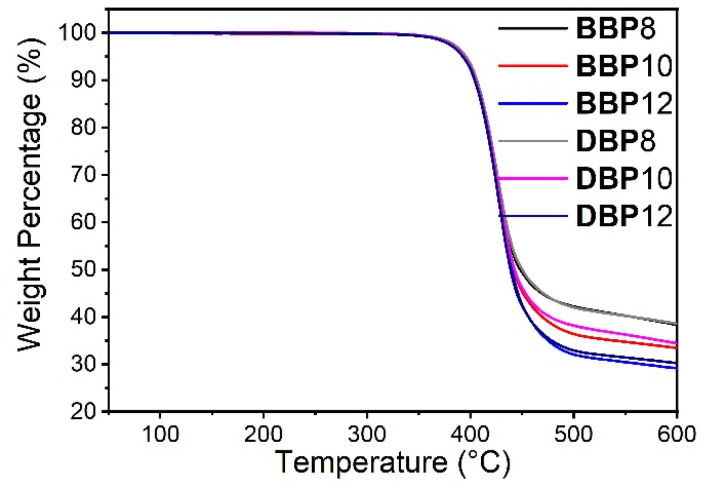
TGA curves for synthesized pyrene-based PAHs.

All these π-extended pyrene-based compounds showed very high thermal stability (decomposition temperatures > 350 °C, for less than 1% weight loss, in dynamic mode), with no impact on the aromatic cores’ topology and the alkyl chain lengths, as measured with thermal gravimetrical analysis (Figure 1 and Table S1, TGA).

**Figure 2:**
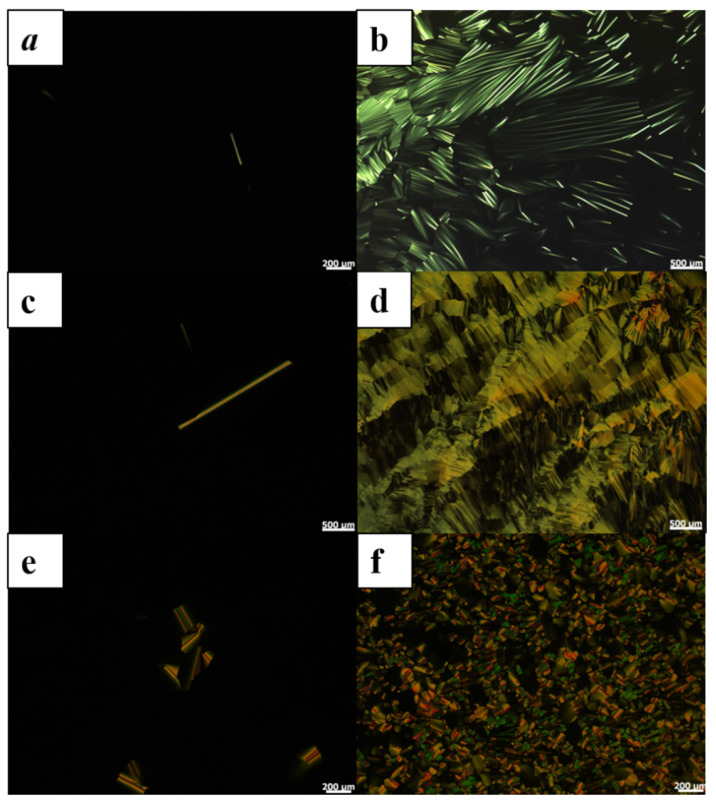
Representative POM photomicrographs (upon cooling from isotropic liquid with a rate of −5 °C/min): (**a**,**b**) **BBP**8, before and after mechanical shearing at 255 °C; (**c**,**d**) **BBP**12, before and after mechanical shearing at 220 °C; (**e**,**f**) **DBP**8, before and after mechanical shearing at 130 °C and 175 °C.

The mesomorphic properties of the **BBP***n* and **DBP***n* compounds were first investigated via polarizing optical microscopy (POM). When the samples were slowly cooled from the isotropic liquid, the formation of a few long linear defects was observed within very large homeotropic zones (Figure 2 and Figure S32), characteristic of columnar mesophases’ optical textures and in agreement with the pseudo-discoid molecular structure. The presence of these large homeotropic areas further indicated that both **BBP***n* and **DBP***n* molecules were orthogonally stacked within long-range ordered columns. **BBP**8, **BBP**12, and **DBP**8 exhibited fine textures upon mechanical shearing only. Quite remarkably, **BBP***n* and **DBP***n* showed an excellent tendency for spontaneous homeotropic alignment in the liquid crystal mesophases, and the shear-controlling orientation of the homeotropic alignment of the discotic columns may represent a truly exceptional feature for their potential use as organic semi-conductive materials with enhanced performances [74].

**Figure 3:**
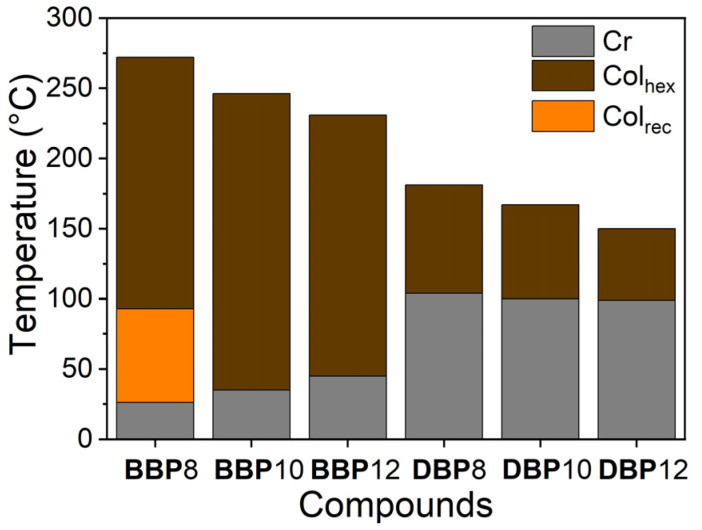
Thermotropic mesophases diagram of **DBP***n* and **BBP***n*; Cr: crystalline phase, Col_rec_: rectangular columnar phase, Col_hex_: hexagonal columnar phase.

The phase transition behavior of the **BBP***n* and **DBP***n* derivatives was analyzed via differential scanning calorimetry (DSC, Figure S31), and the phase transition temperatures and enthalpy changes are listed in Table S2. Both **BBP***n* and **DBP***n* exhibit enantiotropic liquid crystalline phases. As the alkoxy chain length increases, the clearing temperatures are found to decrease stepwise for both series, which, combined with a slight increase in the melting temperatures for **BBP***n* and almost invariance for **DBP***n*, leads to a narrowing of the mesophase ranges in both cases (Figure 3 and Figure S31). **BBP***n* exhibit higher clearing points and wider columnar mesophase ranges than their **DBP***n* isomeric counterparts, with the shortest **BBP***n* homolog, **BBP**8, having the highest clearing point (275 °C) and the widest mesophase range (246 °C). In addition, and as an exception, **BBP**8 displays another phase transformation at a lower temperature, between the solid crystalline state and the mesophase, which, supported by POM observations (Figure S32), corresponds to another columnar mesophase with a different symmetry. Finally, **DBP***n* all exhibited several crystal-to-crystal phase transformations (Table S2).

#### 2.2.2. Mesophase Characterization with SWAXS

**Figure 4:**
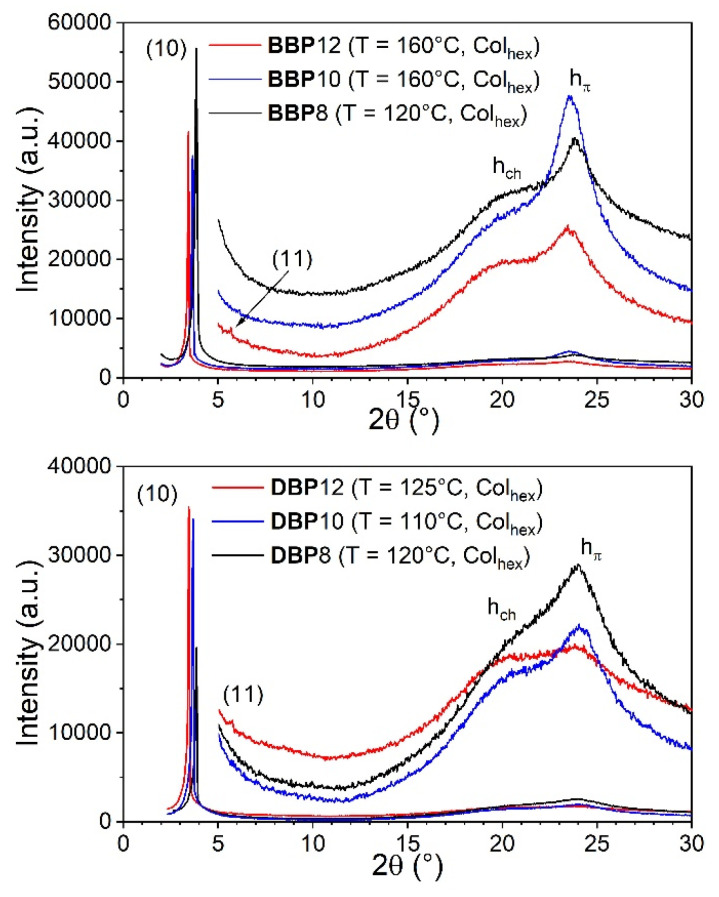
Representative SWAXS patterns of the Col_hex_ mesophases of **BBP***n* (**top**) and **DBP***n* (**bottom**) at different temperatures.

The nature of the mesophases of **DBP***n* and **BBP***n* was ultimately characterized via small- and wide-angle X-ray scattering (SWAXS), and their self-assembly modes within the mesophases were explored (Figure 4 and Figure S33). The main results are reported in Table 1. The SWAXS patterns were not very well developed but presented features characteristic of liquid crystalline mesophases: The octyl and decyl derivatives of both series exhibit only one single but intense and sharp peak in the small angle region, whereas an additional weak reflection could be seen for the dodecyl homologs, with reciprocal spacings in the ratio 1: √3. Supported by their characteristic optical textures (Figure 2 and Figure S32), these features were most readily assigned as the (10) (and (11)) reflection(s) of a hexagonal lattice. These reflections naturally emerge from the segregation between antagonistic domains made of aromatic and aliphatic segments, respectively, and thus define the nature of this interface. The presence of a broad halo with two maxima comprised between ca. 4.3 and 4.5 Å (h_ch_) and between 3.7 and 3.9 Å (h_π_) confirmed the fluid nature of the mesophase, the former signal arising from liquid-like lateral distances between the molten chains (h_ch_), whereas the latter and sharper one resulting from the π–π stacking between consecutive large molecular cores (h_π_). It can be noticed that **BBP***n* homologs have a sharper π–π signal than their isomeric **DBP***n*, indicating stronger and more efficient, and hence, longer-range columnar stacking for the formers. This is fully consistent with DFT calculations (vide infra), which show that the coplanarity of **BBP**1 is slightly better than that of **DBP**1. These observations are reflected in the more extended mesomorphic ranges of **BBP***n* derivatives than for the **DPB***n* ones, consequent to a stronger columnar cohesion. At low temperatures, **BBP**8 further develops a different mesophase with a reduced symmetry, as deduced above by POM and DSC. The SWAXS pattern exhibits seven sharp, low-angle reflections that were indexed into a rectangular symmetry (Table S3). By convention, the group of highest symmetry, *c*2*mm*, corresponding to the centered rectangular lattice is considered; the same wide-angle features as for the Col_hex_ phases above were also displayed (h_ch_ and h_π_).

Therefore, as expected, both **BBP***n* and **DBP***n* regioisomers behave quite differently, attributed mainly to the different molecular symmetry of their cores (*C_2v_* versus *C_2h_*, respectively), which affects (i) the distribution of the peripheral chains around the aromatic cores and (ii) the relative flatness of the central rigid nucleus, and thus, the π–π overlap between neighboring cores along the stacking direction. DFT calculations (vide) performed on the methoxy derivatives (**BBP**1 and **DBP**1) showed different degrees of twisting between the pyrene core and biphenyl part (dihedral angles in Figure S35): For **BBP**1, the twist angles are 20.16 and 19.79°, respectively, whereas for **DBP**1, these twist angles are slightly different, with a value of 21.48° for both angles. Face-to-face interactions between molecules may be affected in some way by this slight divergence from flatness, but they appeared to be more hampered by the chains’ distribution, which was particularly more perturbating in the latter than in the former. Thus, both geometrical parameters are concomitantly likely conductive to stronger intermolecular π–π stackings in **BBP***n* systems than in the **DPB***n* ones and better promoters for the formation of wider and more stable columnar mesophases. A similar observation was made for the isomeric dinaphthopyrene–tetracarboxdiimides (**7** and **8**, Chart 1) [60]. The molecular symmetry (*C_2v_* for **BBP***n* and *C_2h_* for **DBP***n*) has, therefore, a non-negligible effect on both their mesophase ranges and, to some extent, at least at short chain lengths, on the nature of the mesophase with the emergence for one specific case of a Col_rec_ mesophase.

**Figure 5:**
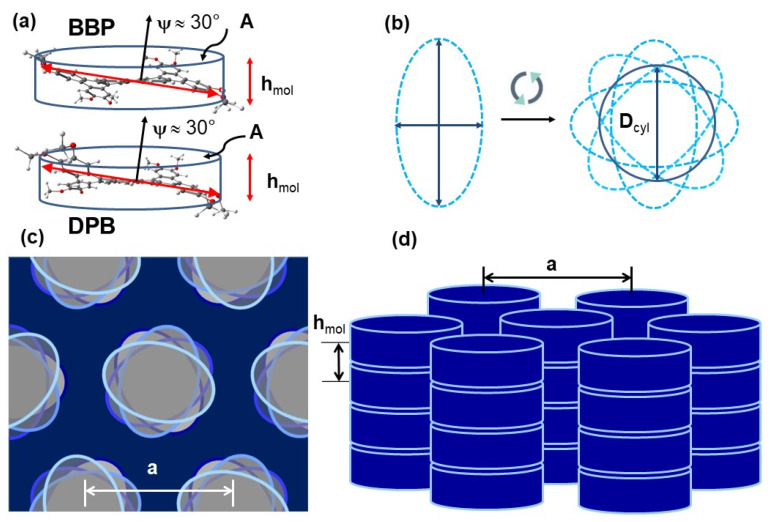
Model for the supramolecular organization in the Col_hex_ phase of **BBP***n* or **DPB***n* molecules: (**a**) the elongated shape of the aromatic core roughly approximates an ellipse of thickness h_mol_ (both **BBP***n* or **DPB***n* molecules substantially deviated from planarity, hence this apparent average tilt ψ, visualized by black arrows, with respect to lattice plane; A, ψ, and h_mol_ defined in Table 1); (**b**) the continuous orientational changes in the stacked cores distributed the space-demanding radiating alkyl chains over the whole stack periphery and design average cylindrical columns of reduced statistical interface area per chain, s_ch_, and average circular diameter, D_cyl_; (**c**) top view and (**d**) side view of the pseudo-cylindrical columns arranged in hexagonal lattice of parameter a and with molecular slice thickness h_mol_ compared with π-stacking distance, h_π_.

As also revealed by DFT calculations (vide infra), the shape of the extended aromatic cores of both isomers deviated from classical disc, which is a priori not ideal with the formation of columnar mesophases organized within hexagonal lattice (Figure 5). Indeed, for the emergence of hexagonal symmetry, columns must have an average circular cross-section and be localized at the nodes of a hexagonal network to allow the homogeneous distribution of the aliphatic chains around the columns into an infinite continuum. Thus here, the core/chain interface, defined by the segregation between the immiscible parts, must take the shape of a cylinder. This is possible if the molecules stack on top of each other with a continuous but random change in the respective orientation of their long molecular axis between neighboring pseudo-ellipsoidal molecules (Figure 5) and possibly some tilts of the cores (ψ, Table 1) with respect to the lattice plane, to generate columns of nearly average circular cross-section (whose resulting equivalent circular cross-sectional diameter can be approximated to D_cyl_; Table 1).

The expansion of the lattice area is similar for both compounds and continuously increases with the alkoxy chain length. The surface area required for the peripheral chains in both series is indeed highly compatible with the interface area offered by these π-conjugated core stacks; the calculated ratio q (Table 1) is very close to unity, indicating that the chains are densely packed around the cores. As mentioned above, the aromatic cores are tilted within the columns, as deduced from the slice thickness, slightly larger than the π–π stacking signals (Table 1). However, the presence of the Col_rec_ mesophase for the shortest homolog results from the decrease in the molecular tilt and likely also from the specific chain distribution (with a side deprived of chains), which likely reduces the molecular rotation around the column axis, leading to columns with a more elliptical cross-section, hence the reduction in the mesophase symmetry.

### 2.3. Photophysical Properties

**Figure 6:**
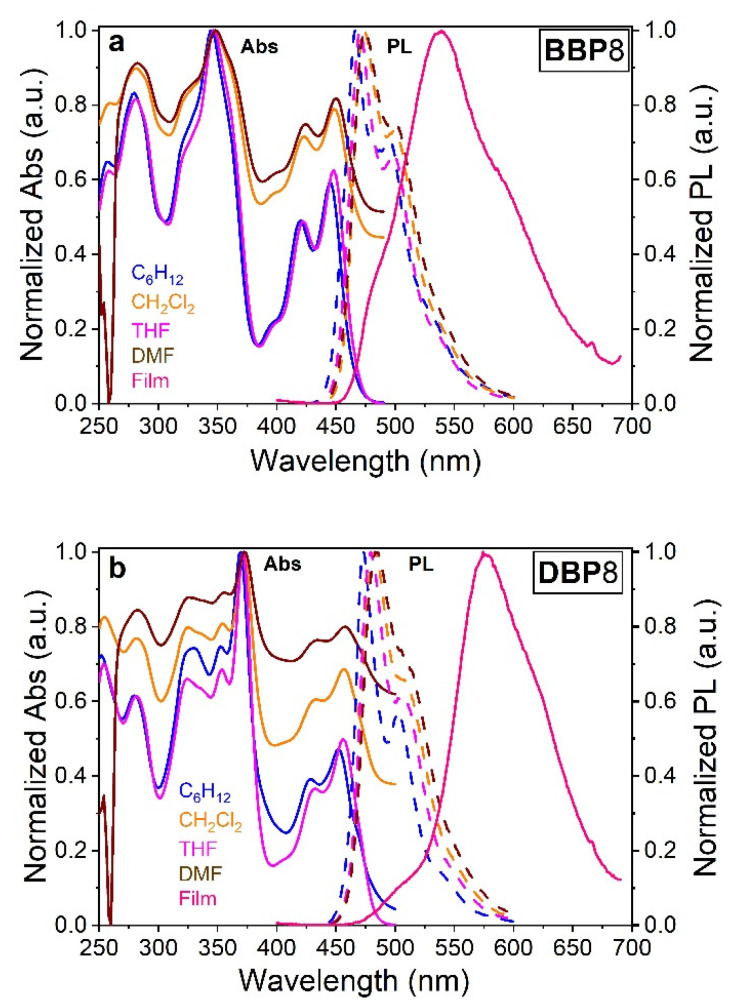
Photophysical properties of **BBP**8 and **DBP**8: UV–Vis absorption, fluorescence emission in solution (c = 1 × 10^−5^ mol/L) in various solvents (cyclohexane, CH_2_Cl_2_, THF = tetrahydrofuran, and DMF = N,N-dimethylformamide) and fluorescence emission in film of (**a**) **BBP**8 and (**b**) **DBP**8.

Pyrene was one of the very first luminescent materials to be studied [1,2] because of its outstanding fluorescent properties, hence the incessant and intense research activity on pyrene-containing compounds for the development of optoelectronic materials. The photophysical properties, i.e., UV–Vis absorption, fluorescence emission, and fluorescence quantum yields, of these new pyrenic systems (**BBP**8 and **DBP**8, chosen as representative compounds) were investigated in different solvents (cyclohexane, dichloromethane, tetrahydrofuran, and N,N-dimethylformamide) in order to explore the effect of solvent polarity on the photophysical properties and in their thin film state (Figure 6, Table 2).

Both **BBP**8 and **DBP**8 exhibited strong and wide absorption bands in the 250–500 nm region, independently of the solvent (Figure 6). **BBP**8 has its maximum absorption at 348 nm, which, according to DFT results of **BBP**1 (Table S5), can be attributed to the contribution of the following frontier molecular orbitals’ electronic transitions: H-−1→L+1(+63%), H→L+3(+15%), H→L+2(+14%). By contrast, for **DBP**8, which has its maximum absorption at 372 nm, it may be attributed to the electronic excitations of H−1→L(+48%), H→L+1(+47%), H-3→L(+16%) (see DFT calculations performed on the methoxy model compounds **BBP**1 and **DBP**1; Figure S34 and Table S5). Thus, the largest wavelength absorptions of both isomers resulted accordingly from their H→L transition.

The maximum absorption peak of **BBP**8 is, therefore, red-shifted by about 24 nm with respect to that of **DBP**8. DFT calculations consistently show that the energy gap between HOMO and LUMO orbitals of **BBP**1 is larger than that of **DBP**1, supporting these experimental measurements. Moreover, **BBP**8 and **DBP**8 also have additional absorption peaks at 448 nm and 456 nm, respectively, mainly corresponding to the H−0→L+0 transition, which is basically consistent with the calculation results on the model compounds (Tables S4 and S5). At smaller wavelengths than the main absorption peak, the overall absorption signal of **DBP**8 appear more structured than that of **BBP**8.

**BBP**8 and **DBP**8 both exhibit yellow-green photoluminescence, with a maximum emission peak spanning between 466–474 nm and 473–483 nm, respectively, depending on the solvent (Figure 6). Fluorescence quantum yields (QYs) measured in the different solvents vary between 17.0% and 31.8% for **BBP**8 and between 22.0% and 36.7% for **DBP**8 (Table 2), and they are found to decrease with the solvent polarity. These effects on UV–Vis, PL, and quantum yield could be interpreted by the interactions of the excited state of the fluorophores with the solvent molecules [76,77]. In the thin-film state, the fluorescence emission ranges of both compounds are wider than that in the solution state, with the single emission peak around 539 nm and 574 nm for **BBP**8 and **DBP**8, respectively. Hence, the maximum emission is strongly red-shifted relative to that in the solution state, of about 65 nm for **BBP**8 and about 90 nm for **DBP**8. This strong difference is attributed to the more effective π orbitals overlap in the film state than in the solution and can be interpreted as the excimer emission [8,26,27,28,45]. According to the results, **BBP***n* and **DBP***n* compounds may be potentially interesting for aggregation-induced emission (AIE) materials and could be used in the fields of fluorescent dyes and fluorescent chemical sensors, as well as electron transport materials in the field of organic optoelectronic semiconductors.

### 2.4. Molecular Aggregation in Solution

**Figure 7:**
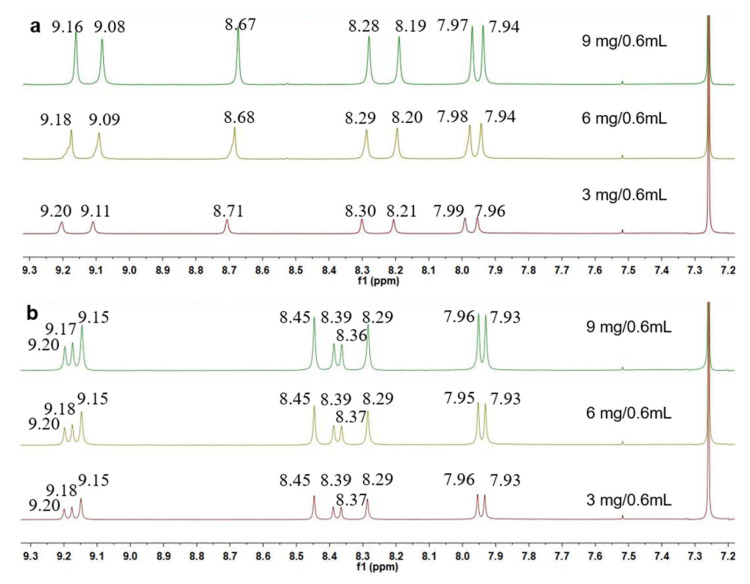
Concentration-dependent ^1^H NMR in CDCl_3_ of (**a**) **BBP**12 and (**b**) **DBP**12.

Polycyclic aromatic hydrocarbons (PAHs) usually develop a strong tendency to aggregate in organic solvents due to the strong core–core interactions between molecules, which depend on the size and coplanarity of the aromatic nuclei of the PAHs. Both **BBP***n* and **DBP***n* possess large conjugated structures that deviate from planarity, which may affect aggregation in organic solvents. Concentration-dependent ^1^H NMR was, therefore, chosen to explore the aggregation behaviors of **BBP**12 and **DBP**12 in CDCl_3_ (Figure 7). All ArH signals shifted slightly to a high field with the increase in the concentration of **BBP**12 (from 3 mg/0.6 mL to 9 mg/0.6 mL, Figure 7a). In other words, with the increase in the concentration, intermolecular π–π face-to-face core interactions are stronger, and molecular aggregation is promoted [78,79], indicating that **BBP***n* isomers have potential application values in the field of gel materials. However, this phenomenon was not observed for the isomeric **DBP**12, i.e., no variation was observed in the protons’ signals with concentration (Figure 7b). These results further demonstrate the important role played by the aromatic core topology and, here, particularly the higher planarity of **BBP**12 with respect to that of **DBP**12 for the aggregation behavior.

### 2.5. DFT Calculations

Density functional theory (DFT) calculations were used to determine the optimized molecular conformations, frontier molecular orbitals’ distributions, energy levels, and band gaps for these two regioisomers. The corresponding methoxy homologs of **BBP***n* and **DBP***n* were selected to simplify the calculations (Figure S34 and Table S4). It was found that both optimized molecular structures of **BBP**1 and **DBP**1 were not perfectly flat, with the biphenyl parts and pyrene skeleton showing some twist angles (Table 3). The coplanarity of **BBP**1 was found to be slightly better than that of **DBP**1, which supports the differences observed in the thermal behavior, SAXS/WAXS, and aggregation in organic solvents of both regioisomers (vide supra).

**Figure 8:**
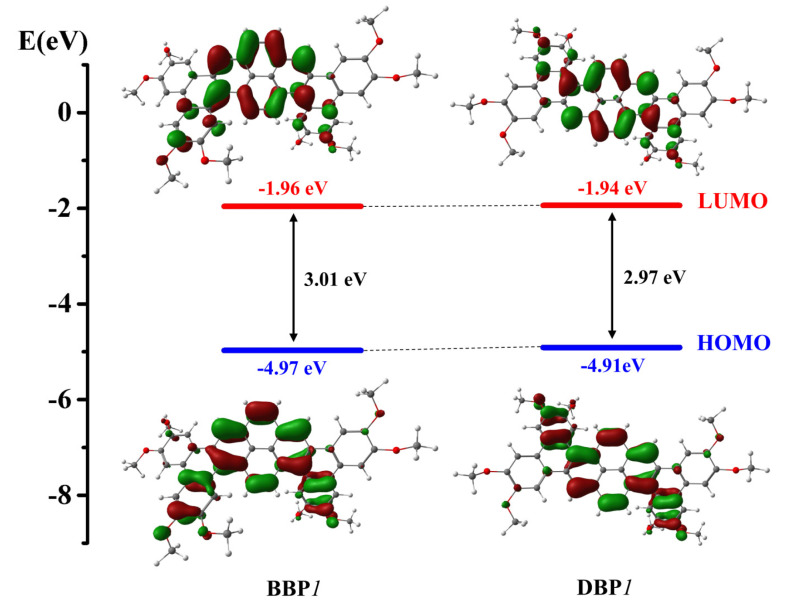
Comparison of HOMO–LUMO energy levels of **BBP**1 and **DBP**1 (isovalues = 0.02), as models for compounds **BBP***n* and **DBP***n*, respectively.

HOMO and LUMO frontiers orbitals are very similar in both cases, as mainly distributed on the pyrene cores in both systems, and thus, as expected, the HOMO–LUMO gaps are also very similar (Figure 8). The HOMO (−4.97 eV) and LUMO (−1.96 eV) of the **BBP**1 lead to a mild wider energy gap (3.01 eV), while for **DBP**1, slightly higher HOMO energy level (−4.91 eV) and higher LUMO level (−1.94 eV) were calculated, and, thus, a narrower energy gap (2.97 eV) (Figure 8 and Figure S36, Table S4). The HOMO–LUMO energy gaps from DFT and experimental UV–Vis absorption spectra thus agree reasonably well: **BBP**1 shows a slightly wider gap value (3.01 eV) than that of **DBP**1, and thus, the corresponding absorption peak of the former is slightly shorter than that of the latter.

## 3. Materials and Methods

Chemicals: All commercially available starting materials were used directly without further purification. The solvents of air- and moisture-sensitive reactions were carefully distilled from appropriate drying agents before use.

Experimental procedures: Air- and moisture-sensitive reactions were assembled on a Schlenk vacuum line or in a glovebox using oven-dried glassware with a Teflon screw cap under an Ar atmosphere. Air- and moisture-sensitive liquids and solutions were transferred using a syringe. Reactions were stirred using Teflon-coated magnetic stir bars. The elevated temperatures were maintained using thermostat-controlled air baths. Organic solutions were concentrated using a rotary evaporator with a diaphragm vacuum pump.

Analytical methods: ^1^H/^13^C-NMR spectra were recorded using a Varian UNITY INOVA 400/100 MHz or Bruker 600 MHz spectrometers in CDCl_3_, and TMS as the internal standard. High-resolution mass spectra (HRMS) spectra were recorded at the Bruker Fourier Transform High-Resolution Mass Spectrometry (solariX XR) with MALDI as the ion source. Elemental analyses (EA) were performed on a Vario Micro Select (Elementar Company, German). The phase transition temperatures and enthalpy changes were investigated using a TA-DSC Q100 differential scanning calorimeter (DSC) under a N_2_ atmosphere with a heating or cooling rate of 10 °C/min. Liquid crystalline optical textures were observed on a polarized optical microscope (POM), namely an Olympus BH2 polarized optical microscope, equipped with a Mettler FP82HT hot stages, the temperatures of which were controlled with XPR-201 and Mettler FP90. SAXS experiments were performed on a Rigaku Smartlab (3) X-ray diffractometer equipped with a TCU 110 temperature control unit. The sample temperature was controlled within ±1 °C. The X-ray sources (Cu Kα, λ = 0.154 nm) were provided using 40 kW ceramic tubes. UV–Vis absorption spectra were recorded on a Perkin Elmer Lambda 950 spectrophotometer at room temperature. Fluorescence was measured on a HORIBA Fluoromax-4p, and the quantum yields were measured with a HORIB-F-3029 Integrating Sphere (HORIBA, Kyoto, Japan). DFT calculations were performed with the B3LYP-D3 method with a base set of 6–311 g (d,p) in Gaussian 09 [80] in the gas phase to obtain the lowest energy conformations of **BBP**1 and **DBP**1 and their HOMO/LUMO distributions and energy levels.

General procedure for the synthesis of **DBP***n* and **BBP***n*: Under the protection of argon, 1,6- or 1,8-dibromopyrene (1.0 equiv.), 4,4,5,5-tetramethyl-2-(3′,4,4′,5-tetrakis(alkoxy)-[1,1′-biphenyl]-2-yl)-1,3,2-dioxaborolane (**3a**/**3b**/**3c**, 2.8 equiv.), K_2_CO_3_ (30.0 equiv.), Pd(PPh_3_)_4_ (20 mol%), and THF/H_2_O (4/1, 0.01 M) were added to a reaction tube. The resulting solutions were stirred at 70 °C for 48 h. The reaction mixtures were cooled to room temperature and extracted with dichloromethane. The residue was preliminarily purified via silica-gel column chromatography, using dichloromethane/petroleum ether (1:2) mixed solvents as the eluent to obtain white solids (**D***n* or **B***n*). Then, a solution of FeCl_3_ (6.0 equiv.) in CH_3_NO_2_ (0.10 M) was added to a stirred solution of **D***n* or **B***n* (1.0 equiv.) in CHCl_3_ (0.0017 M), transferred in a drying tube filled with anhydrous calcium chloride, and the reaction mixtures were stirred at room temperature. The reactions’ progress was tracked every 10 min. After half an hour, the tracking time was reduced to 5 min. After the completion of the reactions was confirmed, methanol was added to terminate the reactions, and the products were extracted with chloroform and distilled water; then, the organic phases were dried with anhydrous MgSO_4_, filtered to remove MgSO_4_, and spin-dried in vacuo. The residues were purified using hot-silica-gel column chromatography, and the eluent was a chloroform and petroleum ether (1:2) mixture; finally, the residues were recrystallized with ethyl acetate and ethanol (5:1) to obtain yellow solids (**DBP***n*/**BBP***n*) with a total yield of 53–64%.

## 4. Conclusions

In summary, two series of π-extended pyrene derivatives bearing flexible alkyl chains of different lengths and differing in the molecular symmetry of the polycyclic aromatic cores were synthesized in good overall yields using the tandem method of Suzuki–Miyaura cross-coupling and Scholl oxidative cyclodehydrogenation reactions, starting from dibromopyrene and appropriate dioxaborolane derivatives. Both sets of isomers formed columnar liquid crystalline mesophases (mainly hexagonal but a rectangular phase was induced for one of the shortest terms) through self-organization. **BBP***n* displayed much broader columnar mesophase ranges with higher thermal stability (higher clearing temperatures) than their **DBP***n* isomeric counterparts, a consequence of the core symmetry, hence the aliphatic chain distribution around the core and their planarity. Both sets also display yellow-green photoluminescence with fluorescence quantum yields between 30% and 40%, modulated by core flatness and chain distribution. DFT calculations were in good agreement with the experimental results and allowed for an understanding and explanation of the difference in their properties. The large homeotropic area alignment behavior of these two types of mesogens on a glass substrate and the column orientation easily controlled by mechanical shearing imply that their potential applications in electronic devices are worth investigating further. Our attempt at understanding the influence of the molecular structure on the π–π interactions and physicochemical properties provides a valid guide to design more alkylated PAHs, with or without heterocyclic constituents, with predictable physical properties and tailorable optoelectronic functions. These isomers could be applied not only in the field of fluorescent dyes but also in the field of organic photoelectric semiconductor materials as electron transport materials. Further investigation of novel DLC isomer systems is currently in progress.

## Figures and Tables

**Table 1:** Compounds and mesophase parameters and geometrical data.

	Cpds	T ^a^	V_mol_^b^	*ρ* ^b^	a/b ^c^	A ^c^	h_mol_^d^	h_ch_	h_π_ (ξ) ^d^	ψ ^e^	χ_core_^f^	D_cyl_^g^	s_ch_^h^	q ^i^
**BBP**8	Col_rec_	87	2505	1.013	53.80/ 24.05	646.95	3.87	4.29	3.71	17	0.265	14.77	22.45	1.00
	Col_hex_	120	2570	0.987	27.03	632.82	4.06	4.32	3.74	23	0.259	14.44	23.04	1.01
	Col_hex_	240	2867	0.885	27.23	641.98	4.46	4.34	3.84	30	0.232	13.77	24.15	0.98
**BBP**10	Col_hex_	80	2943	0.989	28.20	688.60	4.27	4.32	3.70	30	0.226	14.08	23.62	1.06
	Col_hex_	160	3135	0.928	28.39	698.22	4.49	4.39	3.77	33	0.212	13.73	24.21	1.03
	Col_hex_	230	3338	0.872	28.43	700.17	4.77	4.51	3.87	36	0.199	13.32	24.95	1.01
**BBP**12	Col_hex_	75	3382	0.971	30.56	809.04	4.18	4.41	3.71	27	0.197	14.23	23.36	1.05
	Col_hex_	160	3612	0.909	30.67	814.56	4.43	4.37	3.78	29	0.184	13.81	24.03	1.02
	Col_hex_	200	3737	0.878	30.79	821.32	4.55	4.53	3.88	31	0.178	13.64	24.37	1.01
**DPB**8	Col_hex_	120	2570	0.987	26.43	604.93	4.25	4.28	3.71	29	0.259	14.12	23.55	1.03
	Col_hex_	165	2670	0.950	26.47	606.81	4.40	4.27	3.76	31	0.249	13.87	23.97	1.02
**DPB**10	Col_hex_	110	3010	0.967	28.28	692.81	4.34	4.36	3.70	32	0.221	13.96	23.79	1.05
	Col_hex_	150	3109	0.936	28.55	706.06	4.40	4.39	3.72	32	0.214	13.87	23.96	1.03
**DPB**12	Col_hex_	125	3511	0.935	30.38	799.30	4.39	4.32	3.72	32	0.189	13.88	23.93	1.04
	Col_hex_	140	3553	0.924	30.67	814.56	4.36	4.30	3.76	30	0.187	13.93	23.85	1.03

^a^ Temperature of experiment (°C); ^b^ molecular volume (Å^3^) and density (g cm^−3^) calculated from partial volumes of reference substances: V_mol_ = V_ar_ + V_ch_, the sum of the volume of the aromatic part, V_ar_ (from reference compounds), and the volume of the chains, V_ch_ = n_ch_ × (*n*V_CH2_ + ΔV_CH3_), where n_ch_ is the number of chains per molecule, i.e., n_ch_ = 8; *n* is the number of methylene groups per chain; V_CH2_ is the volume of one methylene unit; and ∆V_CH3_ is the volume contribution of the end methylene (as a function of temperature in °C); see reference [75]; *ρ* = MW/(N_A_ × V_mol_); ^c^ lattice parameter, a (Å) and area, A = a^2^√3/2 (Å^2^) for Col_hex_ and a, b, and S = a × b = 2 × A for Col_rec_; ^d^ columnar slice thickness, h_mol_ = V_mol_/A (Å), and stacking distance, h_π_ (Å), average π–π stacking distance between molecules determined from S/WAXS pattern, and h_ch_ is average distance between molten chains; ^e^ out-of-plane tilt angle of mesogen cores inside columns (°), ψ = arcos(h_π_/h_mol_); ^f^ aromatic volume fraction, χ_core_ = V_ar_/V_mol_, with V_ar_ ≈ 665 Å^3^; ^g^ diameter (Å) of equivalent circular cross-sectional area A_core_, D_cyl_ = √(4 × A_core_/π), with A_core_ = χ_core_ × A; ^h^ cylinder area per chain: s_ch_ = πD_cyl_ × h_mol_/n_ch_ (Å^2^); note that the calculation of the interface in the Col_rec_ phase (**BBP**8), the cross-section of the column was considered circular for facilitating the calculation (see references [65,67]); ^i^ chain-packing ratio: q = s_ch_/σ_ch_, σ_ch_ (Å^2^) is the cross-sectional area of a molten chain (see [75]).

**Table 2:** Spectroscopic parameters of **BBP**8 and **DBP**8 in various solvents and thin films ^a^.

	Solution					Film
Compounds	Solvent	λ_abs_	ε	λ_em_	QY	λ_em_
**BBP**8	C_6_H_12_	280 344 420 446	7.40 × 10^4^ 8.90 × 10^4^ 4.36 × 10^4^ 5.25 × 10^4^	466 495	17.0	539
	CH_2_Cl_2_	282 348 422 448	7.83 × 10^4^ 9.58 × 10^4^ 4.66 × 10^4^ 5.95 × 10^4^	474 500	25.2	
	THF	280 346 422 448	8.00 × 10^4^ 9.83 × 10^4^ 4.78 × 10^4^ 6.13 × 10^4^	470 498	26.3	
	DMF	282 348 424 450	6.82 × 10^4^ 8.32 × 10^4^ 4.04 × 10^4^ 5.23 × 10^4^	474 498	31.8	
**DBP**8	C_6_H_12_	252 280 330 352 370 428 452	4.74 × 10^4^ 4.06 × 10^4^ 4.87 × 10^4^ 4.89 × 10^4^ 6.48 × 10^4^ 2.66 × 10^4^ 3.16 × 10^4^	473 503	22.0	574
	CH_2_Cl_2_	254 282 324 354 372 434 456	8.40 × 10^4^ 7.31 × 10^4^ 7.86 × 10^4^ 8.05 × 10^4^ 11.63 × 10^4^ 4.26 × 10^4^ 5.78 × 10^4^	483 508	26.9	
	THF	254 282 326 354 372 432 456	7.90 × 10^4^ 6.91 × 10^4^ 7.41 × 10^4^ 7.72 × 10^4^ 11.29 × 10^4^ 4.10 × 10^4^ 5.61 × 10^4^	479 506	27.8	
	DMF	240 282 326 356 372 434 458	2.94 × 10^4^ 3.15 × 10^4^ 3.50 × 10^4^ 3.64 × 10^4^ 4.78 × 10^4^ 2.31 × 10^4^ 2.68 × 10^4^	483 509	36.7	

^a^ Absorption (λ_abs_) and emission (λ_em_) wavelengths in nm; molecular absorption coefficient (ε, L mol^−1^ cm^−1^); absolute quantum yields, QY, (%) measured in corresponding solvent at 1 × 10^−5^ mol/L, λ_ex_ = 350 nm.

**Table 3:** Ball and stick model of optimized molecular structures of **BBP**1 and **DBP**1. Red: oxygen; dark gray: carbon; light gray: hydrogen.

Compd.	Top Views	Side Views
**BBP**1	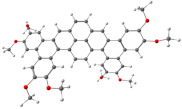	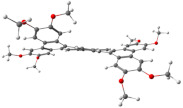
**DBP**1	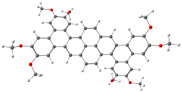	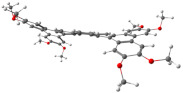

## Data Availability

Not applicable.

## Supplementary Materials

The following supporting information can be downloaded at: https://www.mdpi.com/article/10.3390/molecules28041721/s1, General synthetic procedures and characterization; Scheme S1: Preparation of the biphenylboronic ester derivatives; Figures S1: ^1^H NMR spectrum of **2a**; Figure S2: ^1^H NMR spectrum of **2b**; Figure S3: ^1^H NMR spectrum of **2c**; Figure S4: ^1^H NMR spectrum of **3a**; Figure S5: ^1^H NMR spectrum of **3b**; Figure S6: ^1^H NMR spectrum of **3c**; Figure S7: ^1^H NMR spectrum of **DBP**8; Figure S8: ^1^H NMR spectrum of **DBP**10; Figure S9: ^1^H NMR spectrum of **DBP**12; Figure S10: ^1^H NMR spectrum of **BBP**8; Figure S11: ^1^H NMR spectrum of **BBP**10; Figure S12: ^1^H NMR spectrum of **BBP**12; Figures S13–S18: ^13^C NMR spectrum of **BBP**8, **BBP**10, **BBP**12, **DBP**8, **DBP**10, and **DBP**12; Figures S19–S30: HMRS of **B**8; Figure S20: HMRS of **B**10; Figure S21: HMRS of **B**12; Figure S22: HMRS of **D**8; Figure S23: HMRS of **D**10; Figure S24: HMRS of **D**12; Figure S25: HMRS of **DBP**8; Figure S26: HMRS of **DBP**10; Figure S27: HMRS of **DBP**12; Figure S28: HMRS of **BBP**8; Figure S29: HMRS of **BBP**10; Figure S30: HMRS of **BBP**12; Table S1: Temperatures of decomposition at 1%, 2%, and 5% weight loss for **BBP***n* and **DBP***n*; Table S2: Mesophases, transition temperatures, and enthalpy changes for **DBP**8/10/12 and **BBP**8**/**10/12 (DSC heating/cooling rate is 10 °C/min); Figure S31: DSC traces of **BBP***n* and **DBP***n* at scanning rate of 10 °C/min (heating red, cooling in blue); Figure S32: POM textures of **DBP***n* and **BBP***n* upon cooling from the isotropic liquid; Table S3: Indexation and geometrical parameters of the columnar mesophases of **DBP***n* and **BBP***n*; Figure S33: SAXS patterns of the mesophases of compounds **DBP***n* and **BBP***n* (recorded upon cooling); Figure S34: Molecular structures of model compounds **BBP**1 and **DBP**1; Figure S35: Dihedral angles of compounds **BBP**1 and **DBP**1; Figure S36: Partial molecular orbital diagram for **BBP**1 and **DBP**1 with some selected isodensity (isovalues = 0.02) frontier molecular orbital mainly involved in the electronic transitions; Table S4: List of selected molecular orbital energies for **BBP**1 and **DBP**1 and their HOMO–LUMO energy gaps (ΔE); Table S5: Selected calculated excitation energies (ΔE), oscillator strengths (f), main orbital components, and assignment for the **BBP**1 and **DBP**1 in THF solution.
