# Supplementary material for: Pyrene-Fused Poly-Aromatic Regioisomers: Synthesis, Columnar Mesomorphism, and Optical Properties
Source: Molecules. 2023 Feb 10;28(4):1721. doi: 10.3390/molecules28041721 (PMC9959431; doi:10.3390/molecules28041721)
Supplement: Supplementary file 1 [file molecules-28-01721-s001.zip › molecules-2035953-supplementary.pdf]

Supporting Information

Pyrene-Fused Poly-Aromatic Regioisomers: Synthesis, Columnar Mesomorphism and Physical Properties

Qing Zeng[1], Shuai Liu[1], Hang Lin[1], Ke-Xiao Zhao[1], Xiao-Yan Bai[1], Ke-Qing Zhao[1,*], Ping Hu[1], Bi-Qin Wang[1] and Bertrand Donnio[2,*]

[1] College of Chemistry and Materials Science, Sichuan Normal University, Chengdu, 610066, China

[2] Institut de Physique et Chimie des Materiaux de Strasbourg (IPCMS), CNRS-Université de Strasbourg (UMR 7504), Strasbourg, 67034, France

Contents

1. Synthesis and Characterization

2. ^{1}H NMR

3. ^{13}C NMR

4. HRMS

5. DSC

6. POM

7. SAXS/WAXS

8. DFT

1. Synthesis and Characterization

1.1. Synthesis and characterization of the biphenylboronic ester derivatives (3a/3b/3c)

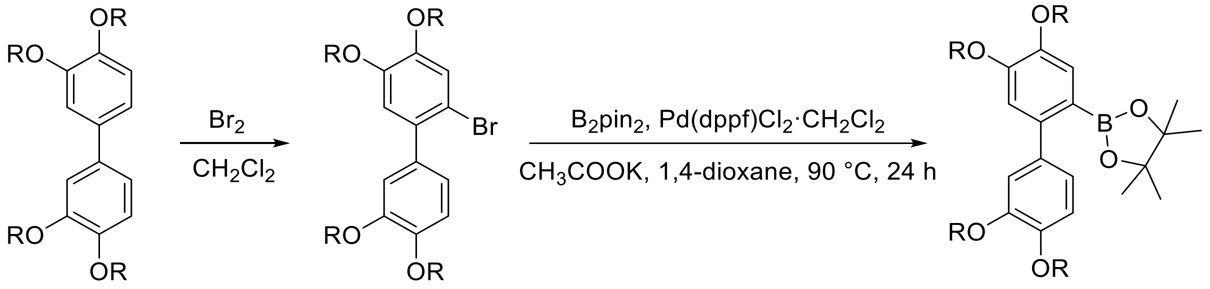

1a, R = C_8H_{17}
1b, R = $C_{10}H_{21}$
1c, R = $C_{12}H_{25}$,

2a, R = C_8H_{17}, 90%
2b, R = $C_{10}H_{21}$, 94%
2c, R = $C_{12}H_{25}$, 97%

3a, R =C_8H_{17}, 82%
3b, R = $C_{10}H_{21}$, 40%
3c, R = $C_{12}H_{25}$, 53%

Scheme S1. Preparation of the biphenylboronic ester derivatives.

1.1.1. 2-Bromo-3',4,4',5-tetrakis(alkoxy)-1,1'-biphenyl (2a/2b/2c)

Compound **1a**/**1b**/**1c** (1.0 equiv.) was dissolved in dry chloroform (0.015 M), bromine (1.0 equiv.) was diluted with chloroform (0.1 M) and added slowly by a constant-pressure dropping funnel. The resulting solution was stirred at room temperature until completion of the reaction. The reaction mixture was quenched with aqueous sodium hydrogen sulfite and extracted with dichloromethane. The combined organic layers were dried over anhydrous $MgSO_4$ and concentrated in vacuo. The crude product was purified by column chromatography on silica gel with dichloromethane/petroleum ether (1/2) mixture as eluent. Finally, recrystallized from ethyl acetate and ethanol gave compounds **2a**/**2b**/**2c** in yield of 90-97%.

2a: According to the general synthesis procedure, **1a** (1.50 g, 2.25 mmol) was converted to the white solid **2a** (1.68 g, 90%). **^{1}H NMR** (400 MHz, $CDCl_3$, TMS) δ 7.10 (s, 1H, ArH), 6.95 (s, 1H, ArH), 6.89 (s, 2H, ArH), 6.84 (s, 1H, ArH), 4.05 - 3.95 (m, 8H, OCH_2), 1.87 - 1.76 (m, 8H, CH_2), 1.48 - 1.43 (m, 8H, CH_2), 1.33 - 1.29 (m, 32H, CH_2), 0.91 - 0.86 (m, 12H, CH_3).

2b: According to the general synthesis procedure, **1b** (0.63 g, 0.81 mmol) was converted to the white solid **2b** (0.65 g, 94%). **^{1}H NMR** (400 MHz, $CDCl_3$, TMS) δ 7.11 (s, 1H, ArH), 6.95 (s, 1H, ArH), 6.90 (s, 2H, ArH), 6.84 (s, 1H, ArH), 4.05 - 3.95 (m, 8H, OCH_2), 1.86 - 1.78 (m, 8H, CH_2), 1.48 - 1.27 (m, 56H, CH_2), 0.90 - 0.86 (m, 12H, CH_3).

2c: According to the general synthesis procedure, **1c** (3.29 g, 3.70 mmol) was converted to the white solid **2c** (3.50 g, 97%). **^{1}H NMR** (400 MHz, $CDCl_3$, TMS) δ 7.10 (s, 1H, ArH), 6.94 (s, 1H, ArH), 6.89 (s, 2H, ArH), 6.84 (s, 1H, ArH), 4.04 - 3.95 (m, 8H, OCH_2), 1.87 - 1.75 (m, 8H, CH_2), 1.50 - 1.42 (m, 8H, CH_2), 1.36 - 1.26 (m, 64H, CH_2), 0.89 - 0.86 (m, 12H, CH_3).

1.1.2. 4,4,5,5-Tetramethyl-2-(3',4,4',5-tetrakis(alkoxy)-[1,1'-biphenyl]-2-yl)-1,3,2-dioxaborolane (3a/3b/3c)

Compound **2a**/**2b**/**2c** (1.0 equiv.), bis(pinacolato)diboron (1.5 equiv.), CH_3COOK (3.0 equiv.), $Pd(dppf)Cl_2{\cdot}CH_2Cl_2$ (5 mol%), 1,4-dioxane (0.06 M) were added in a round bottom flask. The resulting solution was stirred under nitrogen at 90 ºC for 24 h. The reaction mixture was cooled to room temperature and extracted with dichloromethane. The combined organic layers were

dried over anhydrous $MgSO_4$ and concentrated in vacuo. The crude product was purified by column chromatography on silica gel with dichloromethane/petroleum ether (1/3) mixture as eluent. Finally, recrystallized from ethyl acetate and methanol gave **3a**/**3b**/**3c** in yield of 40-82%.

3a: According to the general synthesis procedure, **2a** (4.00 g, 5.37 mmol) was converted to the white solid **3a** (3.51 g, 82%). **^{1}H NMR** (400 MHz, $CDCl_3$, TMS) δ 7.20 (s, 1H, ArH), 6.92 (s, 1H, ArH), 6.85 (s, 3H, ArH), 4.06 - 3.99 (m, 8H, OCH_2), 1.83 - 1.80 (m, 8H, CH_2), 1.47 - 1.46 (m, 8H, CH_2), 1.38 - 1.28 (m, 32H, CH_2), 1.19 (s, 12H, CH_3), 0.92 - 0.88 (m, 12H, CH_3).

3b: According to the general synthesis procedure, **2b** (1.20 g, 1.40 mmol) was converted to the white solid **3b** (1.27 g, 40%). **^{1}H NMR** (400 MHz, $CDCl_3$, TMS) δ 7.21 (s, 1H, ArH), 6.92 (s, 1H, ArH), 6.85 (s, 3H, ArH), 4.06 - 4.00 (m, 8H, OCH_2), 1.85 - 1.78 (m, 8H, CH_2), 1.52 - 1.27 (m, 56H, CH_2), 1.20 (s, 12H, CH_3), 0.90 - 0.86 (m, 12H, CH_3).

3c: According to the general synthesis procedure, **2c** (2.60 g, 2.70 mmol) was converted to the white solid **3c** (1.44 g, 53%). **^{1}H NMR** (400 MHz, $CDCl_3$, TMS) δ 7.21 (s, 1H, ArH), 6.92 (s, 1H, ArH), 6.85 (s, 3H, ArH), 4.05 - 4.00 (m, 8H, OCH_2), 1.85 - 1.80 (m, 8H, CH_2), 1.50 - 1.26 (m, 72H, CH_2), 1.20 (s, 12H, CH_3), 0.90 - 0.87 (m, 12H, CH_3).

1.2. Synthesis and characterization of the DBP*n* and BBP*n*

D8: **HRMS** (ESI) calcd for $C_{104}H_{154}O_8$ $[M]^{+\bullet}$ m/z: 1532.1677 (100.0%), 1531.1644 (88.9%), 1533.1711 (55.7%), 1534.1744 (20.5%), 1535.1778 (5.6%), 1536.1811 (1.2%); found: 1532.1667 (100%), 1531.1635 (87.8%), 1533.1700 (59.1%), 1534.1734 (23.9%), 1535.1770 (6.5%), 1536.1808 (1.5%).

D10: **HRMS** (ESI) calcd for $C_{120}H_{186}O_8$ $[M]^{+\bullet}$ m/z: 1756.4181 (100.0%), 1755.4148 (77.0%), 1757.4215 (64.4%), 1758.4248 (27.4%), 1759.4282 (8.7%), 1760.4315 (2.2%); found: 1756.4185 (100%), 1755.4150 (76.6%), 1757.4217 (69.0%), 1758.4252 (30.6%), 1759.4286 (8.3%), 1760.4320 (2.8%).

D12: **HRMS** (ESI) calcd for $C_{136}H_{218}O_8$ $[M]^{+\bullet}$ m/z: 1980.6685 (100.0%), 1981.6719 (73.0%), 1979.6652 (68.0%), 1982.6752 (35.3%), 1983.6786 (12.7%), 1984.6819 (3.6%); found: 1980.6688 (100%), 1981.6715 (79.4%), 1979.6658 (67.5%), 1982.6746 (40.9%), 1983.6782 (16.7%), 1984.6821 (5.0%).

B8: **HRMS** (ESI) calcd for $C_{104}H_{154}O_8$ $[M]^{+\bullet}$ m/z: 1532.1677 (100.0%), 1531.1644 (88.9%), 1533.1711 (55.7%), 1534.1744 (20.5%), 1535.1778 (5.6%), 1536.1811 (1.2%); found: 1532.1660 (100%), 1531.1629 (86.4%), 1533.1692 (58.6%), 1534.1727 (23.6%), 1535.1762 (6.8%), 1536.1801 (1.8%).

B10: **HRMS** (ESI) calcd for for $C_{120}H_{186}O_8$ $[M]^{+\bullet}$ m/z: 1756.4181 (100.0%), 1755.4148 (77.0%), 1757.4215 (64.4%), 1758.4248 (27.4%), 1759.4282 (8.7%), 1760.4315 (2.2%); found: 1756.4177 (100%), 1755.4145 (77.2%), 1757.4211 (66.7%), 1758.4247 (30.2%), 1759.4280 (9.8%), 1760.4315 (2.7%).

B12: **HRMS** (ESI) calcd for $C_{136}H_{218}O_8$ $[M]^{+\bullet}$ m/z: 1980.6685 (100.0%), 1981.6719 (73.0%), 1979.6652 (68.0%), 1982.6752 (35.3%), 1983.6786 (12.7%), 1984.6819 (3.6%); found: 1980.6695 (100%), 1981.6731 (77.3%), 1979.6666 (67.2%), 1982.6764 (36.7%), 1983.6806 (14.4%), 1984.6826 (4.8%).

DBP8: According to the general synthesis procedure, the substrate 1,6-dibromopyrene (28.5 mg, 0.079 mmol) was converted into a yellow solid **DBP**8 (72.2 mg, 60%). **^{1}H NMR** (400 MHz, $CDCl_3$, TMS) δ 9.18 (d, *J* = 9.2 Hz, 2H, ArH), 9.14 (s, 2H, ArH), 8.44 (s, 2H, ArH), 8.37 (d, *J* = 9.4 Hz, 2H, ArH), 8.28 (s, 2H, ArH), 7.95 (s, 2H, ArH), 7.93 (s, 2H, ArH), 4.36 - 4.29 (m, 12H, OCH_2), 4.23 (t, *J* = 6.5 Hz, 4H, OCH_2), 2.02 - 1.92 (m, 16H, CH_2), 1.61 - 1.55 (m, 16H, CH_2), 1.45 - 1.25 (m, 64H, CH_2), 0.93 - 0.86 (m, 24H, CH_3). **^{13}C NMR** (100 MHz, $CDCl_3$, TMS) δ 149.78, 149.34, 149.15, 147.77, 128.84, 127.52, 127.13, 127.08, 126.86, 125.50, 124.97, 124.68, 124.52, 123.83, 123.78, 117.89, 114.66, 107.94, 107.45, 107.08, 69.72, 69.54, 69.45, 31.89, 29.53, 29.48, 29.38, 29.35, 26.22, 26.19, 22.73, 22.70, 14.16. **Elemental Analysis** ($C_{104}H_{150}O_8$, MW 1528.34, %): calc. C 81.73, H 9.89; found C 81.85, H 9.55. **HRMS** (ESI) calcd for $C_{104}H_{150}O_8$ $[M]^{+\bullet}$ m/z: 1528.1364 (100.0%), 1527.1331 (88.9%), 1529.1398 (57.4%), 1530.1431 (20.5%); found: 1528.1332 (100.0%), 1527.1301 (81.0%), 1529.1354 (59.5%), 1530.1372 (24.0%).

DBP10: According to the general synthesis procedure, the substrate 1,6-dibromopyrene (26.8 mg, 0.074 mmol) was converted into a yellow solid **DBP**10 (74.7 mg, 57%). **^{1}H NMR** (600 MHz, $CDCl_3$, TMS) δ 9.18 (d, *J* = 9.2 Hz, 2H, ArH), 9.15 (s, 2H, ArH), 8.44 (s, 2H, ArH), 8.38 (d, *J* = 9.3 Hz, 2H, ArH), 8.28 (s, 2H, ArH), 7.95 (s, 2H, ArH), 7.93 (s, 2H, ArH), 4.36 - 4.30 (m, 12H, OCH_2), 4.23 (t, *J* = 6.5 Hz, 4H, OCH_2), 2.04 - 1.93 (m, 16H, CH_2), 1.65 - 1.54 (m, 16H, CH_2), 1.47 - 1.25 (m, 96H, CH_2), 0.91-0.84 (m, 24H, CH_3). **^{13}C NMR** (100 MHz, $CDCl_3$, TMS) δ 149.79, 149.36, 149.17, 147.80, 128.86, 127.54, 127.16, 127.10, 126.89, 125.52, 124.99, 124.70, 124.54, 123.85, 123.80, 117.91, 114.69, 107.98, 107.48, 107.12, 69.74, 69.57, 69.46, 31.97, 31.93, 29.75, 29.68, 29.64, 29.60, 29.58, 29.55, 29.50, 29.44, 29.39, 26.25, 26.20, 22.74, 22.71, 14.17, 14.14. **Elemental Analysis** ($C_{120}H_{182}O_8$, MW 1752.77, %): calc. C 82.23, H 10.47; found C 82.01, H 10.34. **HRMS** (ESI) calcd for $C_{120}H_{182}O_8$ $[M]^{+\bullet}$ m/z: 1752.3868 (100.0%), 1751.3835 (77.0%), 1753.3902 (64.3%), 1754.3935 (27.4%), 1755.3969 (7.6%); found: 1752.3857 (100.0%), 1751.3825 (72.9%), 1753.3905 (68.7%), 1754.3948 (35.4%), 1755.3996 (12.5%).

DBP12: According to the general synthesis procedure, the substrate 1,6-dibromopyrene (35.1 mg, 0.097 mmol) was converted into a yellow solid **DBP**12 (101.5 mg, 53%). **^{1}H NMR** (400 MHz, $CDCl_3$, TMS) δ 9.18 (d, *J* = 9.2 Hz, 2H, ArH), 9.14 (s, 2H, ArH), 8.45 (s, 2H, ArH), 8.37 (d, *J* = 9.3 Hz, 2H, ArH), 8.28 (s, 2H, ArH), 7.95 (s, 2H, ArH), 7.92 (s, 2H, ArH), 4.36 - 4.29 (m, 12H, OCH_2), 4.24 (t, *J* = 6.4 Hz, 4H, OCH_2), 2.01 - 1.92 (m, 16H, CH_2), 1.62 - 1.54 (m, 16H, CH_2), 1.46 - 1.24 (m, 128H, CH_2), 0.91 - 0.84 (m, 24H, CH_3). **^{13}C NMR** (100 MHz, $CDCl_3$, TMS) δ 149.78, 149.35, 149.15, 147.79, 128.85, 127.53, 127.14, 127.09, 126.88, 125.51, 124.98, 124.69, 124.52, 123.84, 123.79, 117.89, 114.66, 107.96, 107.47, 107.09, 69.76, 69.73, 69.55, 69.45, 31.96, 31.92, 29.78, 29.72, 29.68, 29.60, 29.58, 29.55, 29.48, 29.42, 29.39, 26.24, 26.20, 22.73, 22.69, 14.16, 14.13. **Elemental Analysis** ($C_{136}H_{214}O_8$, MW 1977.20, %): calc. C 82.62, H 10.91; found C 82.69, H 10.72. **HRMS** (ESI) calcd for $C_{136}H_{214}O_8$ $[M]^{+\bullet}$ m/z: 1976.6372 (100.0%), 1977.6406 (73.0%), 1975.6339 (68.0%), 1978.6439 (35.8%); found: 1976.6398 (100.0%), 1977.6444 (88.0%), 1975.6359 (63.0%), 1978.6502 (51.0%).

BBP8: According to the general synthesis procedure, the substrate 1,8-dibromopyrene (27.2 mg, 0.076 mmol) was converted into a yellow solid **BBP**8 (74.1 mg, 64%). **^{1}H NMR** (400 MHz, $CDCl_3$, TMS) δ 9.12 (s, 2H, ArH), 9.05 (s, 2H, ArH), 8.64 (s, 2H, ArH), 8.26 (s, 2H, ArH), 8.17 (s, 2H, ArH), 7.93 (d, *J* = 11.5 Hz, 4H, ArH), 4.38 - 4.27 (m, 16H, OCH_2), 2.04-1.94 (m, 16H,

CH_2), 1.68 - 1.61 (m, 16H, CH_2), 1.50 - 1.27 (m, 64H, CH_2), 0.95 - 0.85 (m, 24H, CH_3). **^{13}C NMR** (100 MHz, $CDCl_3$, TMS) δ 149.60, 149.22, 149.12, 147.68, 129.48, 127.86, 127.02, 126.60, 125.87, 125.43, 124.78, 124.51, 124.29, 123.80, 123.63, 118.87, 114.12, 107.53, 106.93, 106.79, 69.65, 69.54, 69.46, 69.15, 31.94, 31.92, 29.80, 29.73, 29.61, 29.60, 29.54, 29.47, 29.45, 29.43, 26.59,26.30, 26.28, 22.76, 22.72, 14.19, 14.18, 14.13. **Elemental Analysis** ($C_{104}H_{150}O_8$, MW 1528.34, %): calc. C 81.73, H 9.89; found C 81.79, H 9.75. **HRMS** (ESI) calcd for $C_{104}H_{150}O_8$ $[M]^{+\bullet}$ m/z: 1528.1364 (100.0%), 1527.1331 (88.9%), 1529.1398 (57.4%), 1530.1431 (20.5%), 1531.1465 (4.6%); found: 1528.1332 (100.0%), 1527.1330 (83.0%), 1529.1361 (57.0%), 1530.1389 (23.9%), 1531.1449 (7.0%).

BBP10: According to the general synthesis procedure, the substrate 1,8-dibromopyrene (41.8 mg, 0.116 mmol) was converted into a yellow solid **BBP**10 (114.1 mg, 56%). **^{1}H NMR** (400 MHz, $CDCl_3$, TMS) δ 9.14 (s, 2H, ArH), 9.07 (s, 2H, ArH), 8.65 (s, 2H, ArH), 8.27 (s, 2H, ArH), 8.19 (s, 2H, ArH), 7.94 (d, J = 12.4 Hz, 4H, ArH), 4.33 (m, 16H, OCH_2), 2.01 - 1.96 (m, 16H, CH_2), 1.63 - 1.57 (m, 16H, CH_2), 1.45 - 1.25 (m, 96H, CH_2), 0.90 - 0.85 (m, 24H, CH_3). **^{13}C NMR** (100 MHz, $CDCl_3$, TMS) δ 149.64, 149.23, 149.14, 147.73, 129.52, 127.86, 127.04, 126.64, 125.90, 125.45, 124.79, 124.55, 124.32, 123.80, 123.67, 118.87, 114.17, 107.56, 107.01, 106.83, 69.66, 69.56, 69.48, 69.17, 31.99, 31.95,29.82, 29.81, 29.78, 29.74, 29.72, 29.70, 29.66, 29.64, 29.57, 29.53, 29.45, 26.57, 26.31, 26.28, 22.75, 22.72, 14.17, 14.13. **Elemental Analysis** ($C_{120}H_{182}O_8$, MW 1752.77, %): calc. C 82.23, H 10.47; found C 81.93, H 10.30. **HRMS** (ESI) calcd for $C_{120}H_{182}O_8$ $[M]^{+\bullet}$ m/z: 1752.3868 (100.0%), 1751.3835 (77.0%), 1753.3902 (64.3%), 1754.3935 (27.4%), 1755.3969 (7.6%); found: 1752.3867 (100.0%), 1753.3909 (75.0%), 1751.3826 (67.0%), 1754.3951 (35.4%), 1755.3991 (12.5%).

BBP12: According to the general synthesis procedure, the substrate 1,8-dibromopyrene (37.6 mg, 0.105 mmol) was converted into a yellow solid **BBP**12 (111.8 mg, 54%). **^{1}H NMR** (400 MHz, $CDCl_3$, TMS) δ 9.14 (s, 2H, ArH), 9.07 (s, 2H, ArH), 8.66 (s, 2H, ArH), 8.27 (s, 2H, ArH), 8.18 (s, 2H, ArH), 7.94 (d, J = 12.3 Hz, 4H, ArH), 4.37 - 4.29 (m, 16H, OCH_2), 2.05 - 1.92 (m, 16H, CH_2), 1.67 - 1.58 (m, 16H, CH_2), 1.47 - 1.23 (m, 128H, CH_2), 0.91 - 0.84 (m, 24H, CH_3). **^{13}C NMR** (100 MHz, $CDCl_3$, TMS) δ 149.64, 149.23, 149.14, 147.73, 129.53, 127.87, 127.06, 126.65, 125.91, 125.45, 124.80, 124.56, 124.33, 123.81, 123.67, 118.87, 114.17, 107.56, 107.00, 106.81, 69.65, 69.56. 69.46, 69.17, 31.97, 29.80, 29.78, 29.75, 29.67, 29.64, 29.57, 29.52, 29.44, 26.57, 26.31, 26.28, 22.73, 22.72, 14.16,14.14. **Elemental Analysis** ($C_{136}H_{214}O_8$, MW 1977.20, %): calc. C 82.62, H 10.91; found C 82.90, H 10.78. **HRMS** (ESI) calcd for $C_{136}H_{214}O_8$ $[M]^{+\bullet}$ m/z: 1976.6372 (100.0%), 1977.6406 (73.0%), 1975.6339 (68.0%); found: 1976.6375 (100.0%), 1977.6385 (74.0%), 1975.6348 (60.0%).

2. ^{1}H NMR

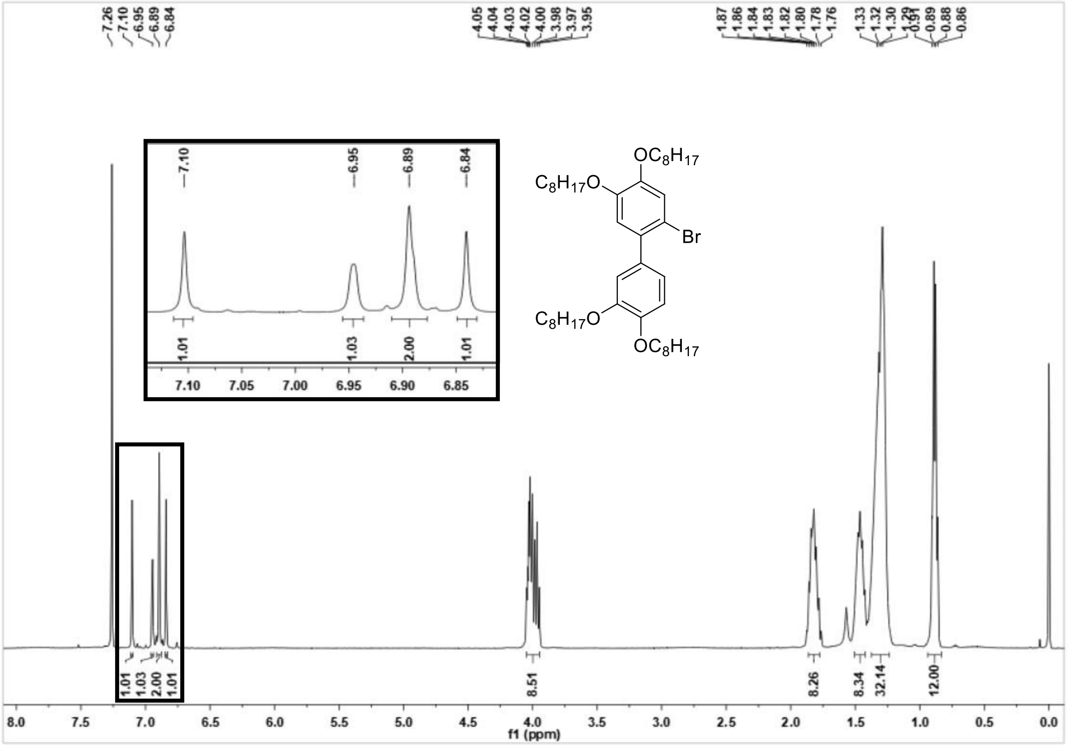

Figure S1. ^{1}H NMR spectrum of **2a**.

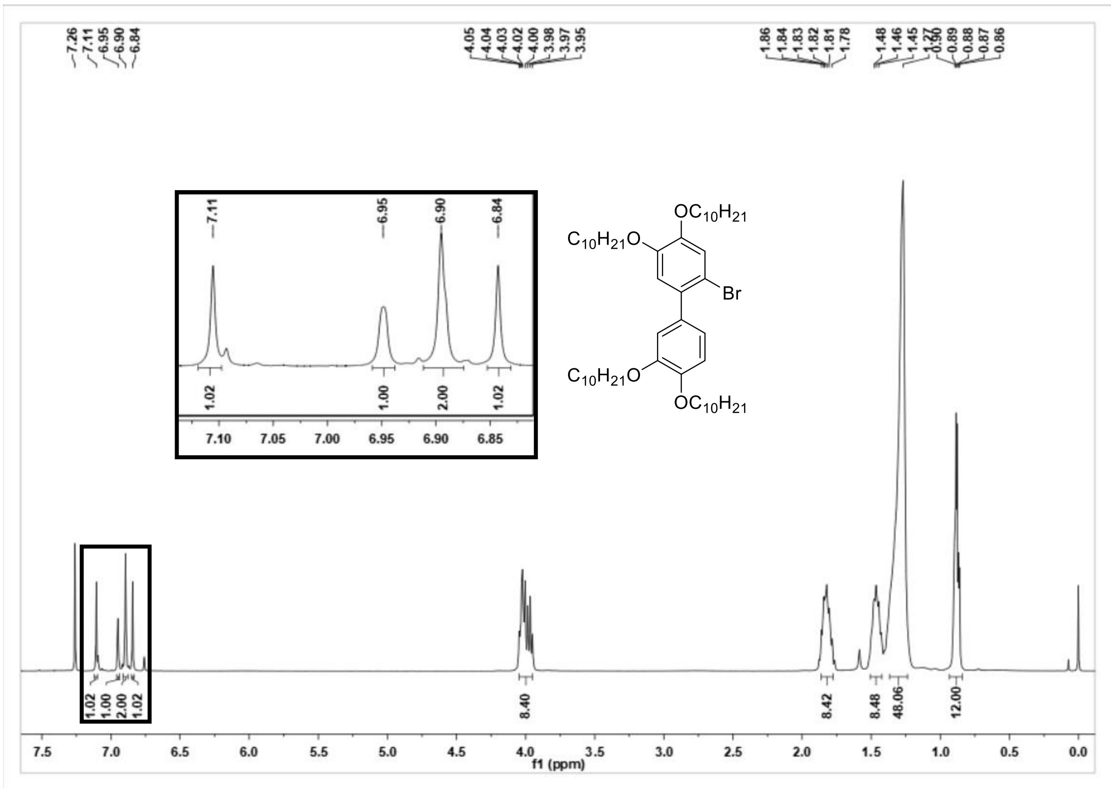

Figure S2. ^{1}H NMR spectrum of **2b**.

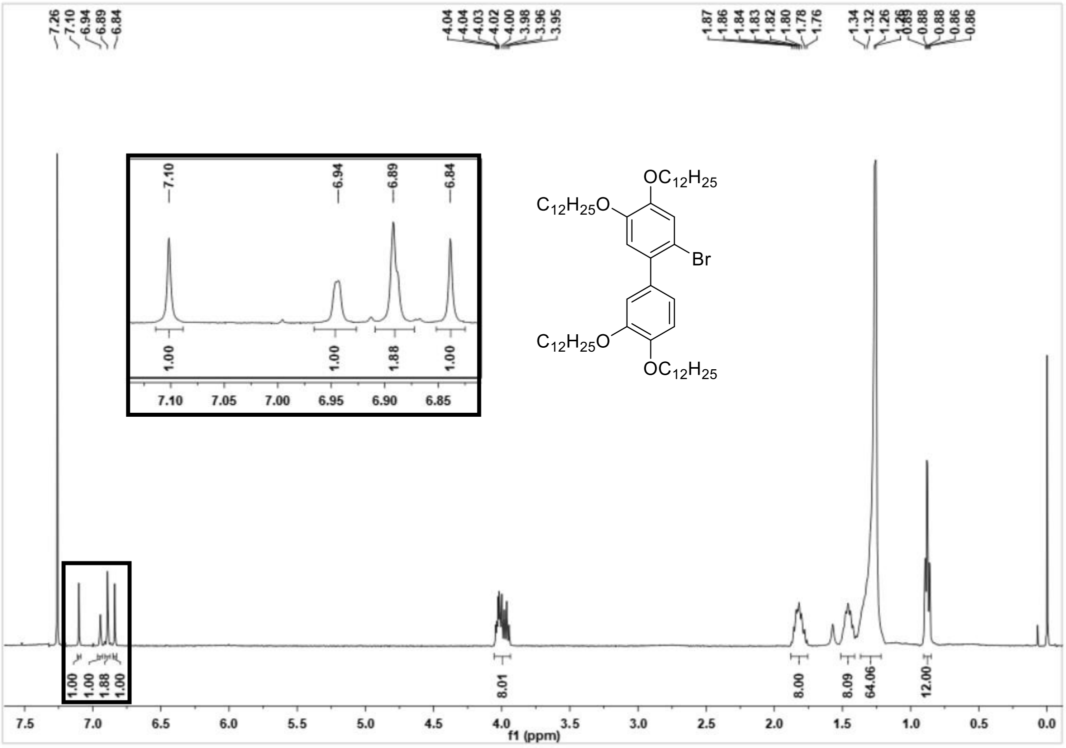

Figure S3. ^{1}H NMR spectrum of **2c**.

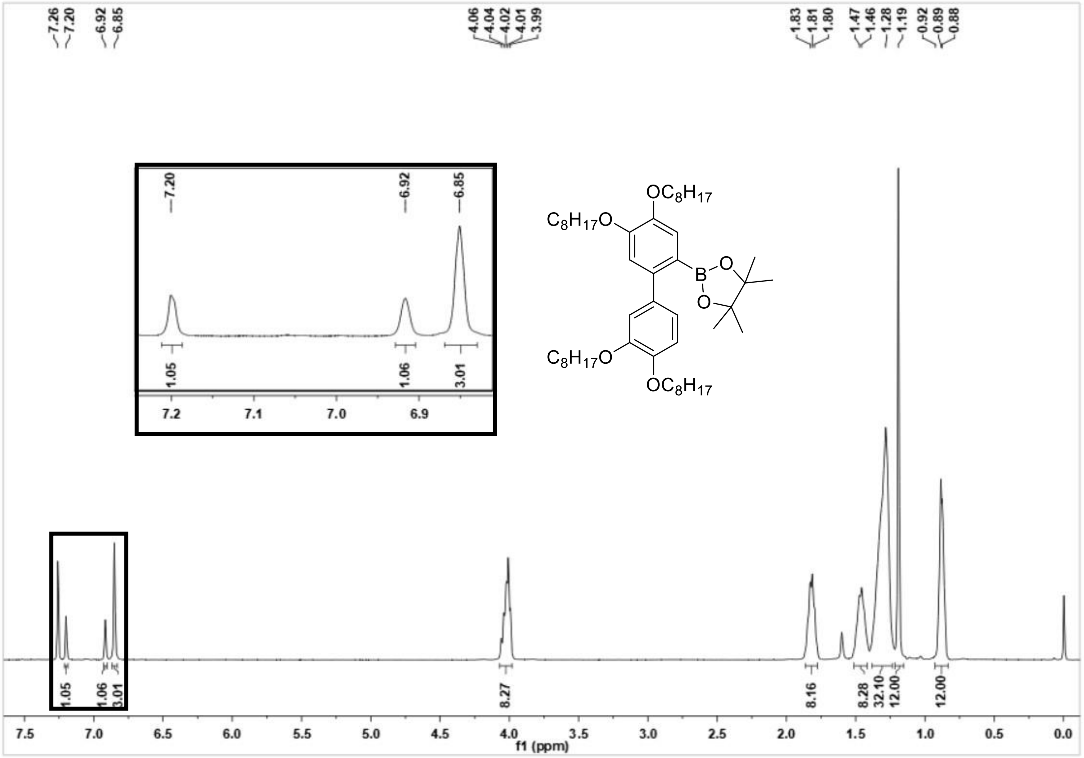

Figure S4. ^{1}H NMR spectrum of **3a**.

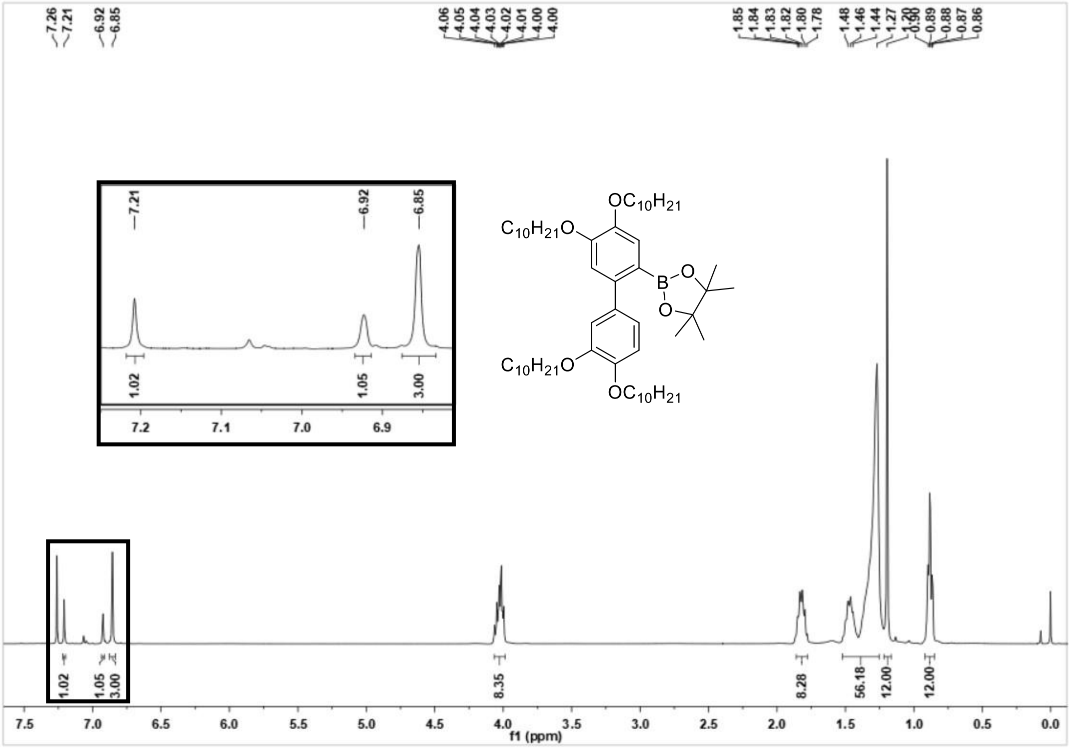

Figure S5. ^{1}H NMR spectrum of **3b**.

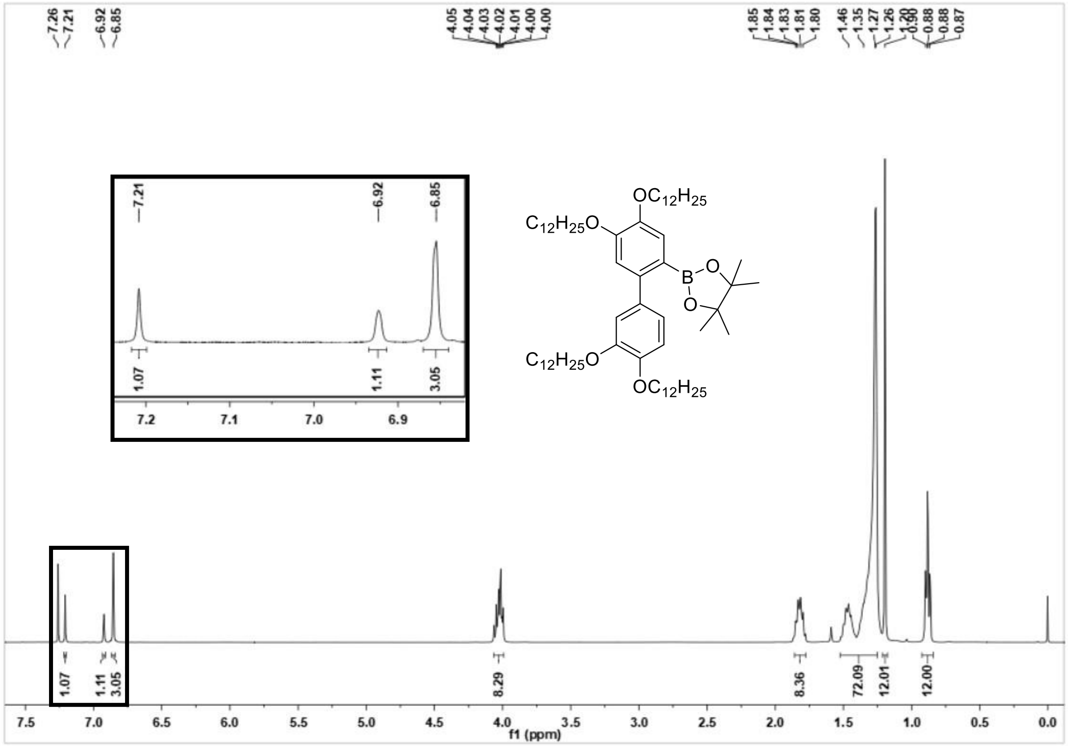

Figure S6. ^{1}H NMR spectrum of **3c**.

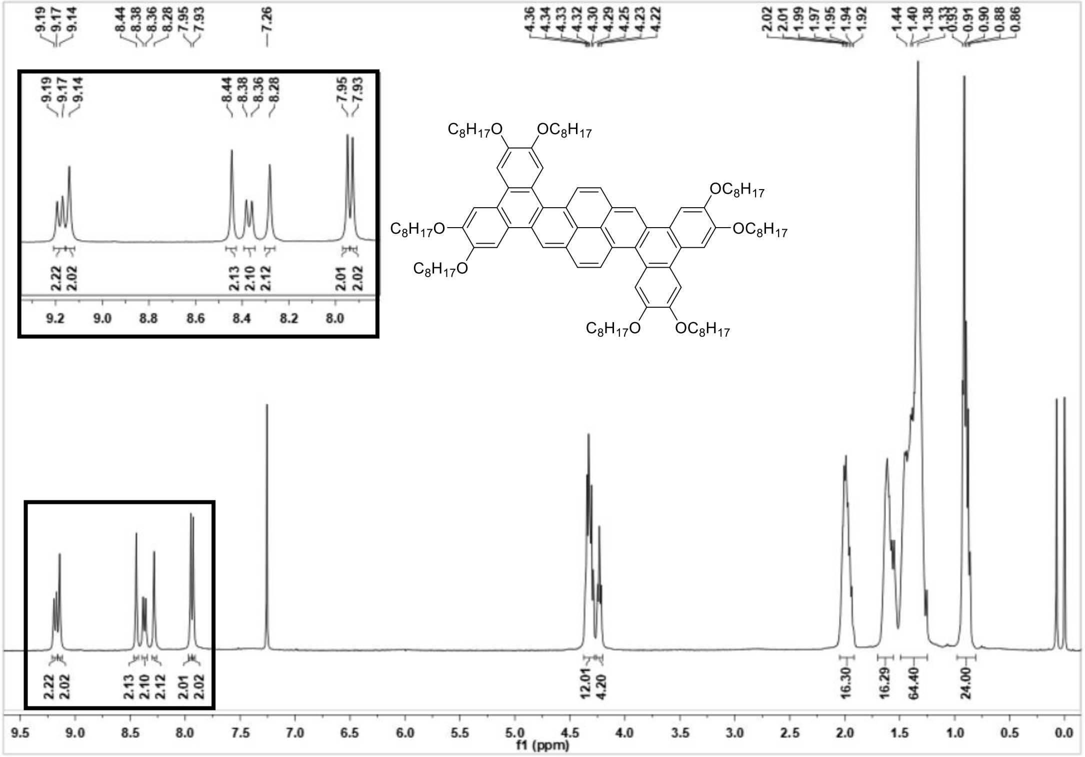

Figure S7. ^{1}H NMR spectrum of **DBP**8.

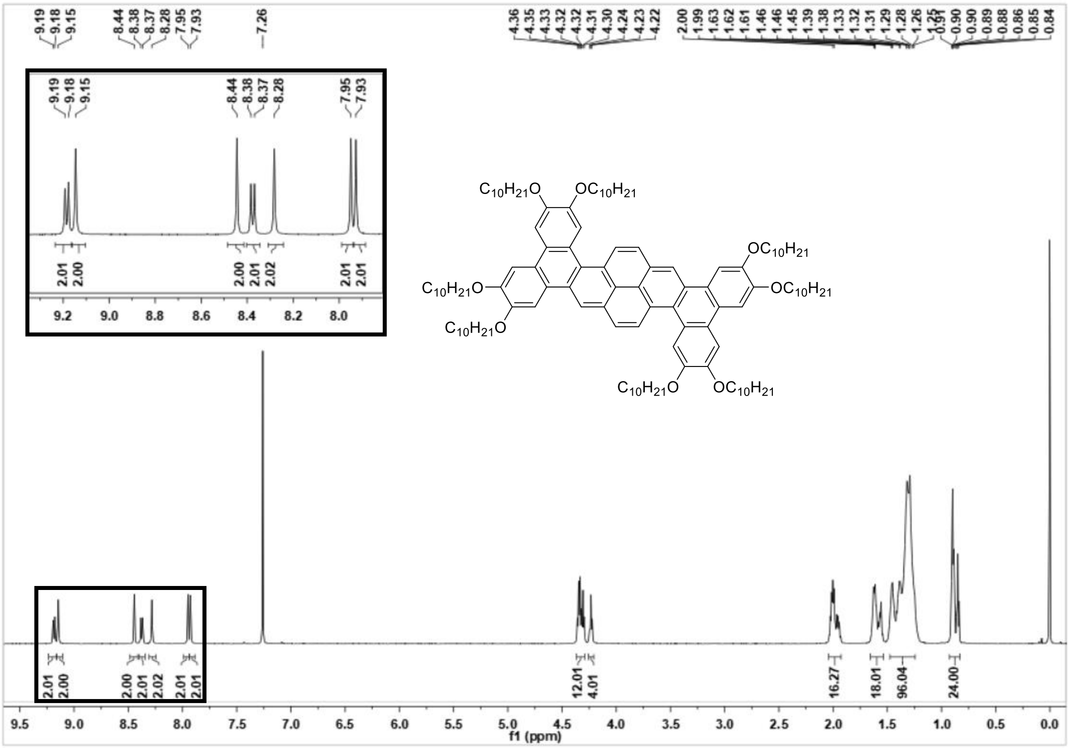

Figure S8. ^{1}H NMR spectrum of **DBP**10.

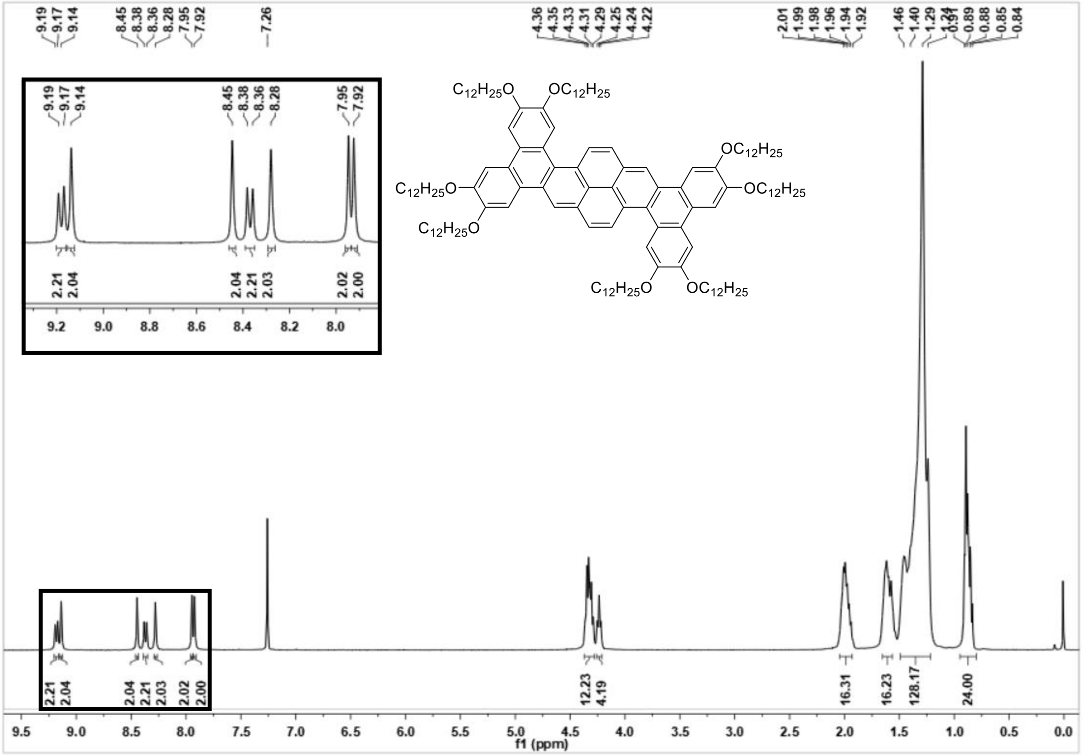

Figure S9. ^{1}H NMR spectrum of **DBP**12.

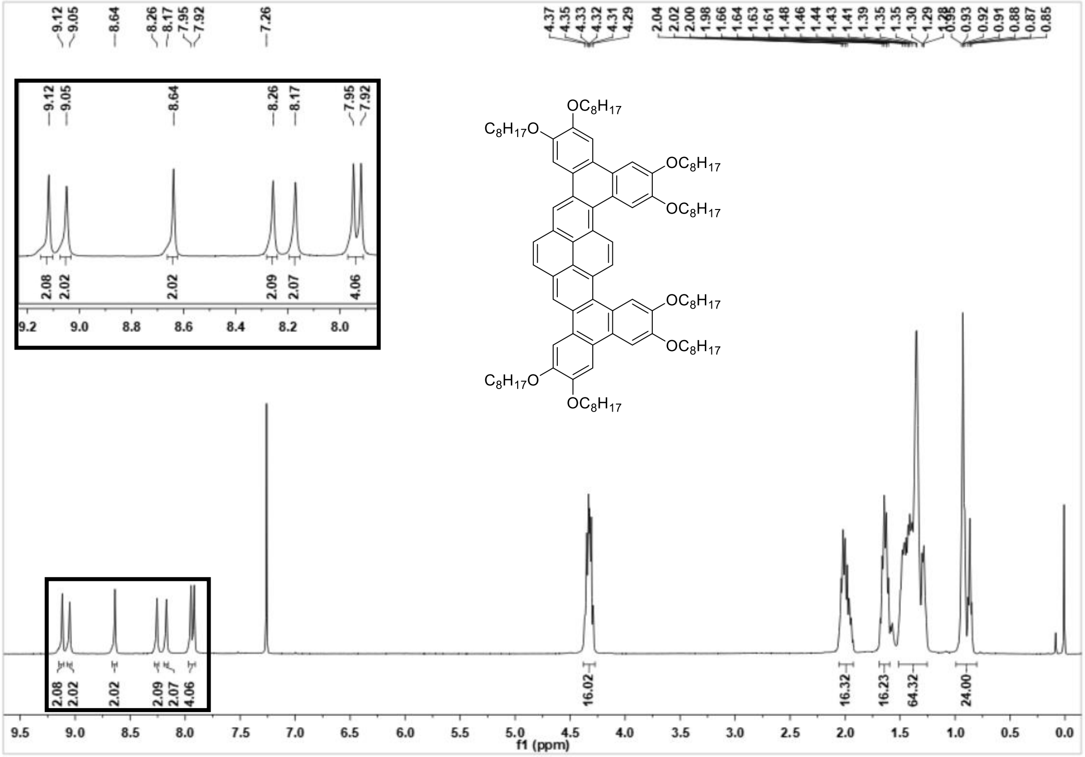

Figure S10. ^{1}H NMR spectrum of **BBP**8.

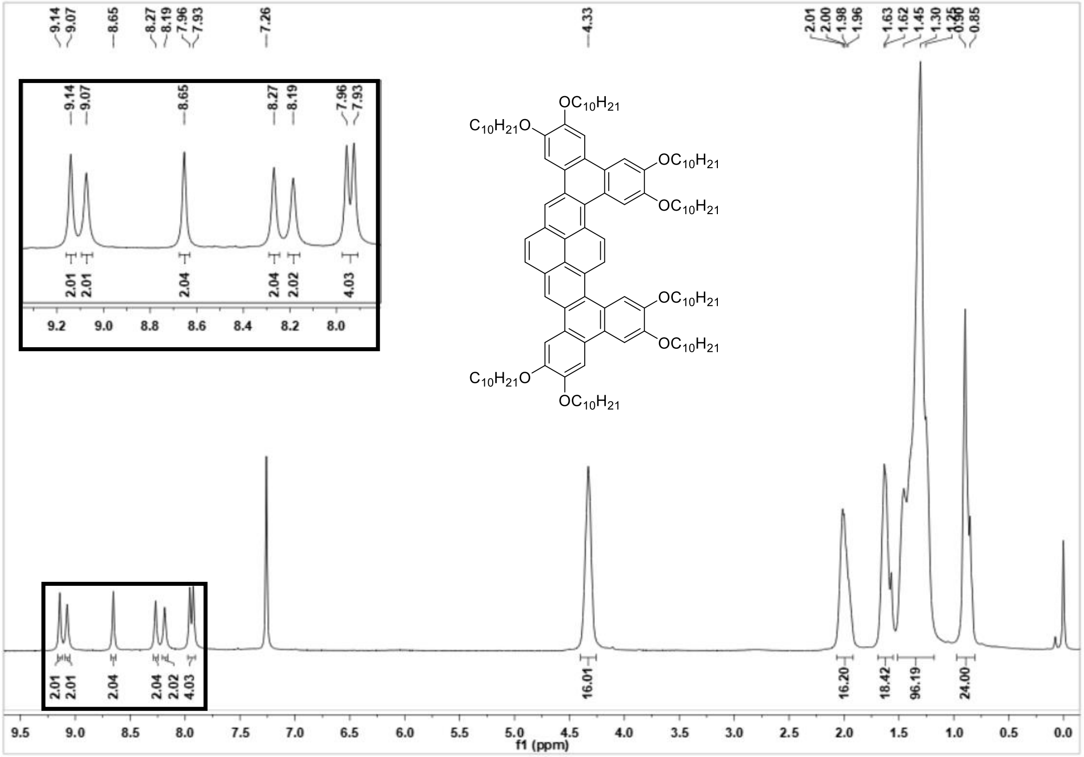

Figure S11. ^{1}H NMR spectrum of **BBP**10.

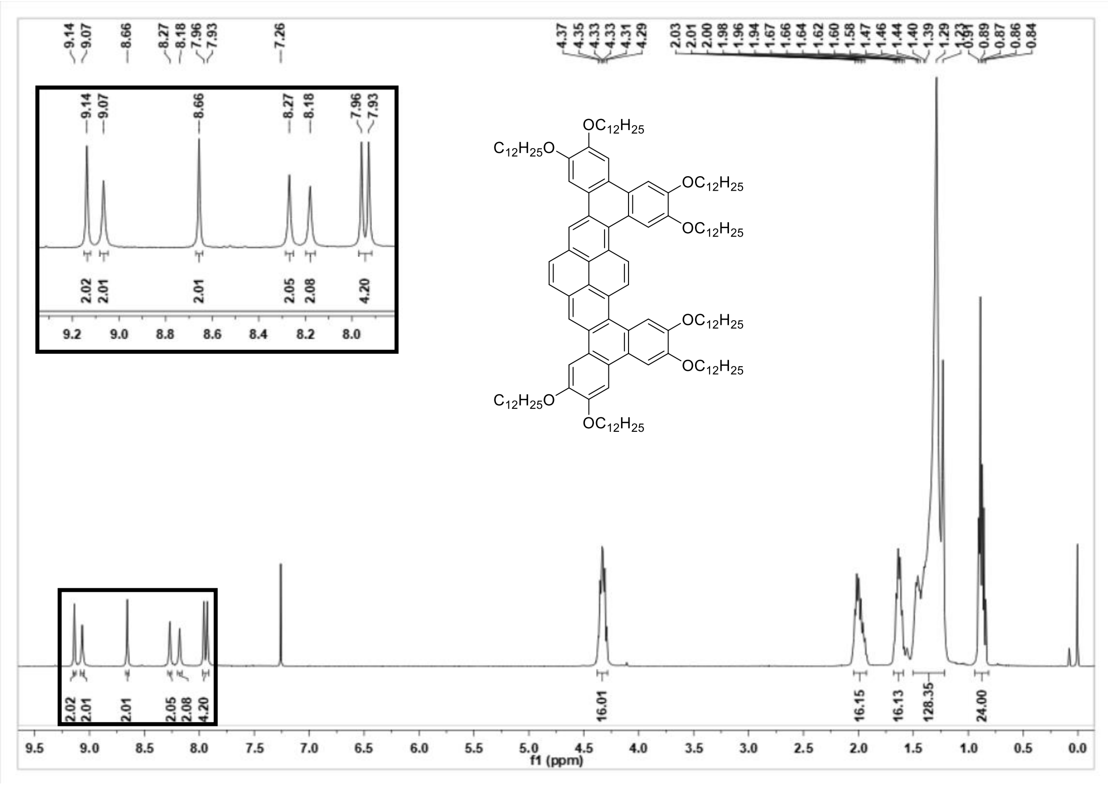

Figure S12. ^{1}H NMR spectrum of **BBP**12.

3. ^{13}C NMR

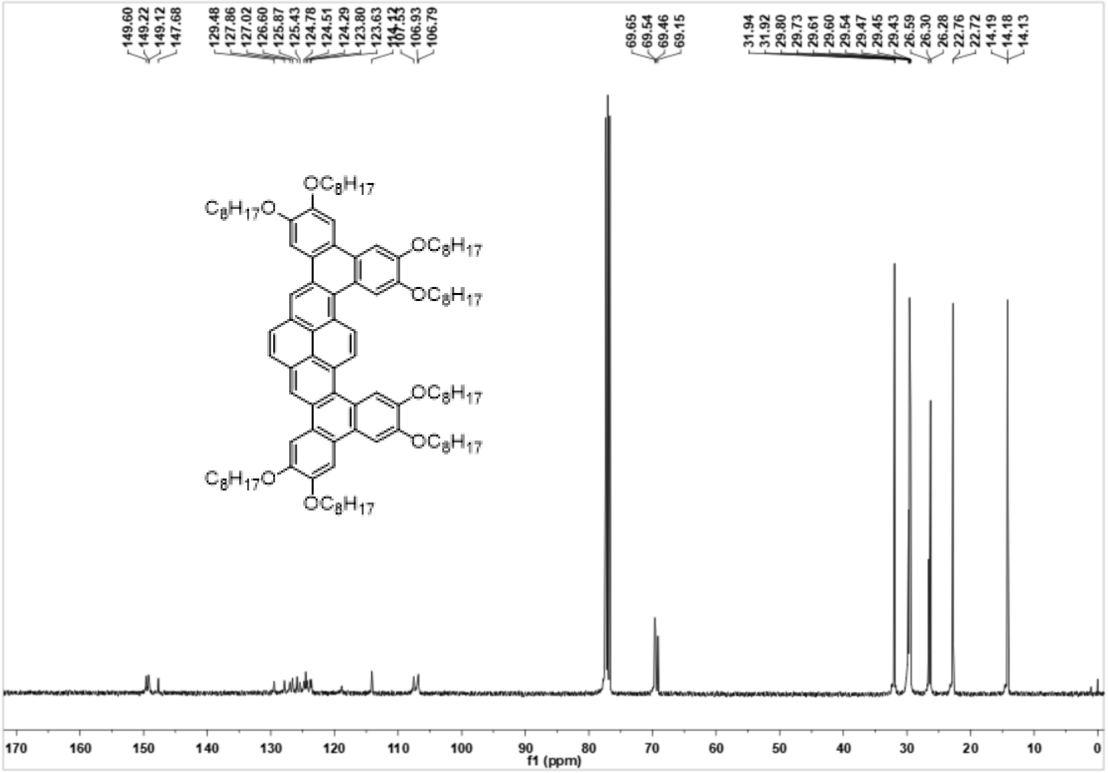

Figure S13. ^{13}C NMR spectrum of **BBP**8.

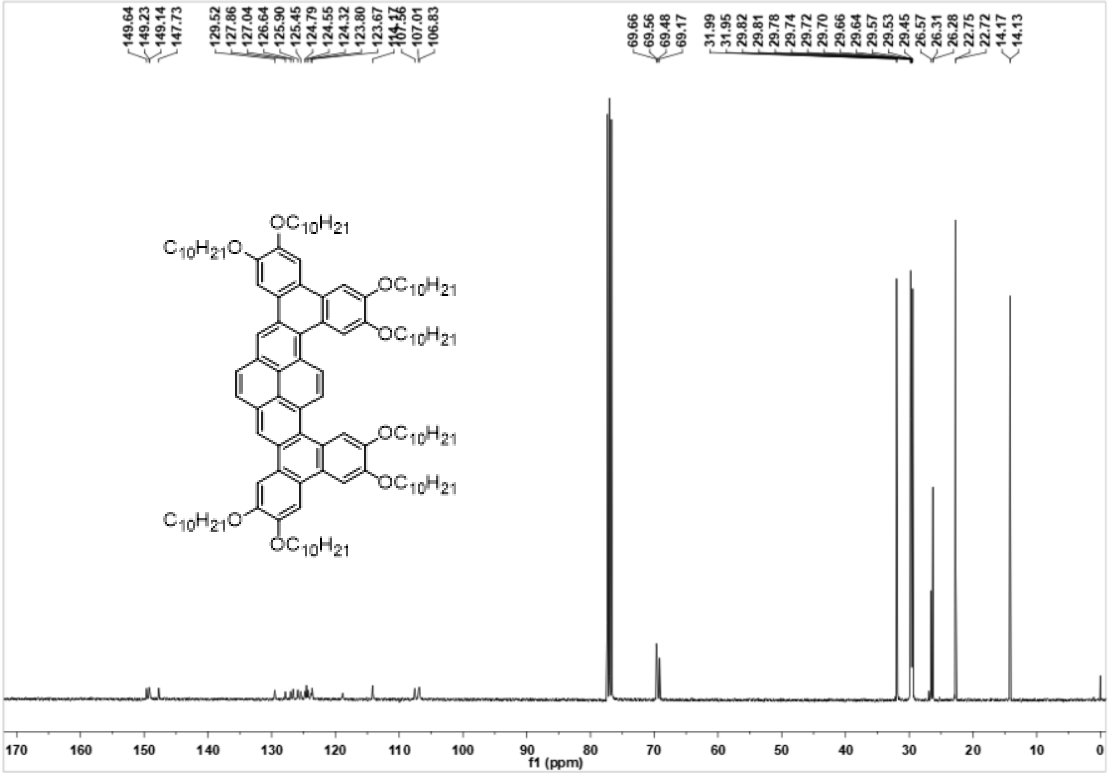

Figure S14. ^{13}C NMR spectrum of **BBP**10.

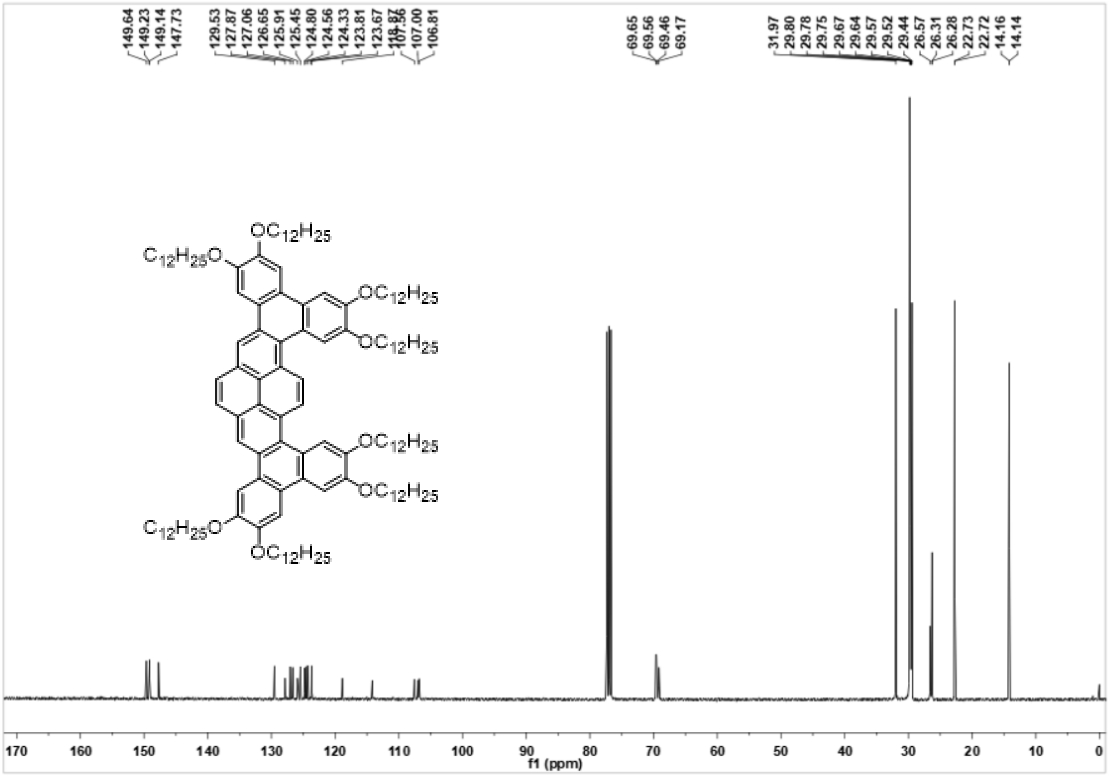

Figure S15. ^{13}C NMR spectrum of **BBP**12.

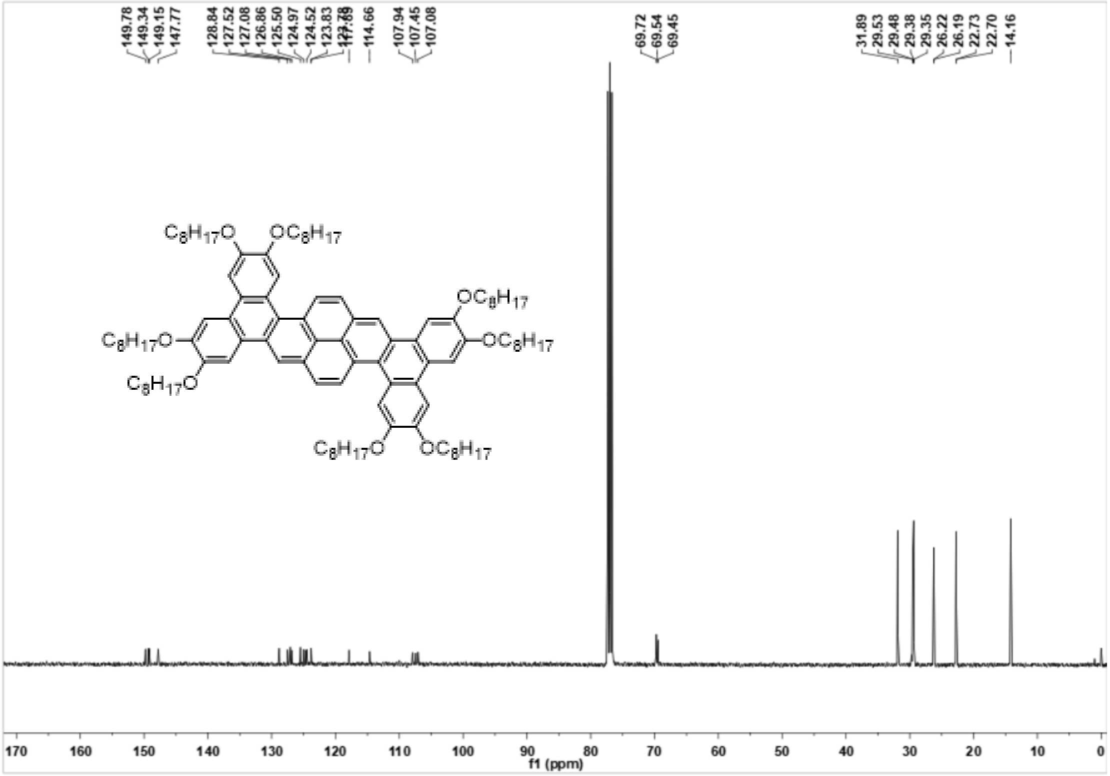

Figure S16. ^{13}C NMR spectrum of **DBP**8.

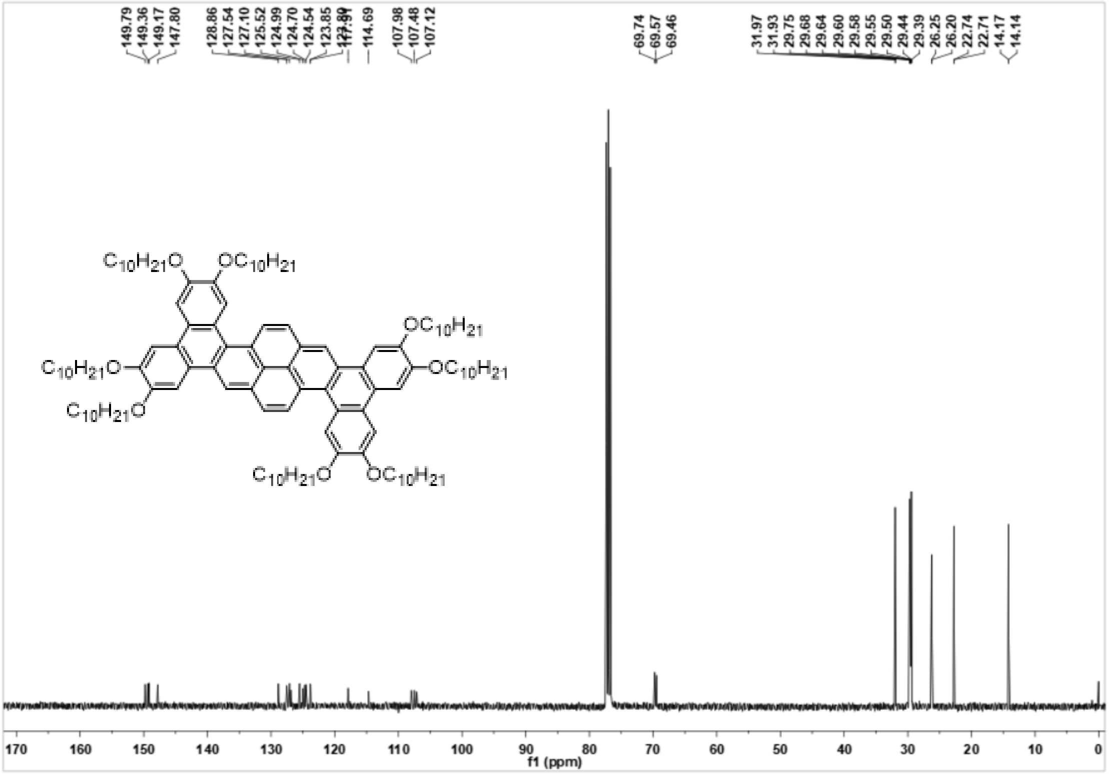

Figure S17. ^{13}C NMR spectrum of **DBP**10.

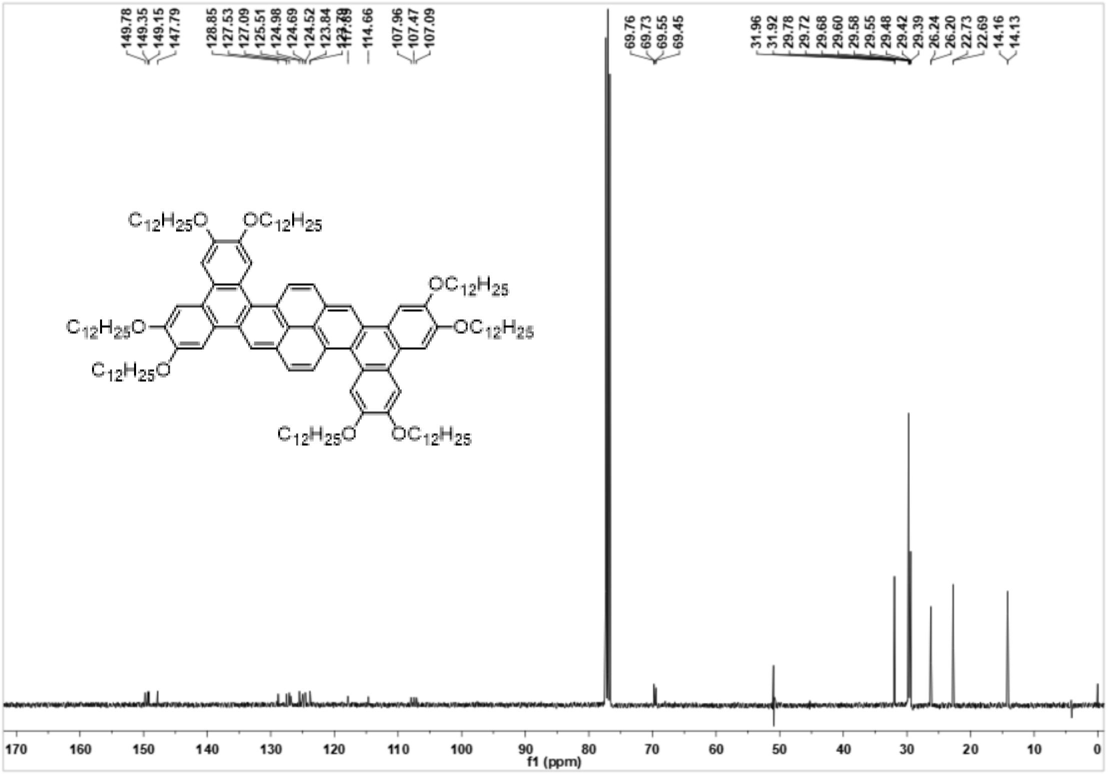

Figure S18. ^{13}C NMR spectrum of **DBP**12.

4. HRMS

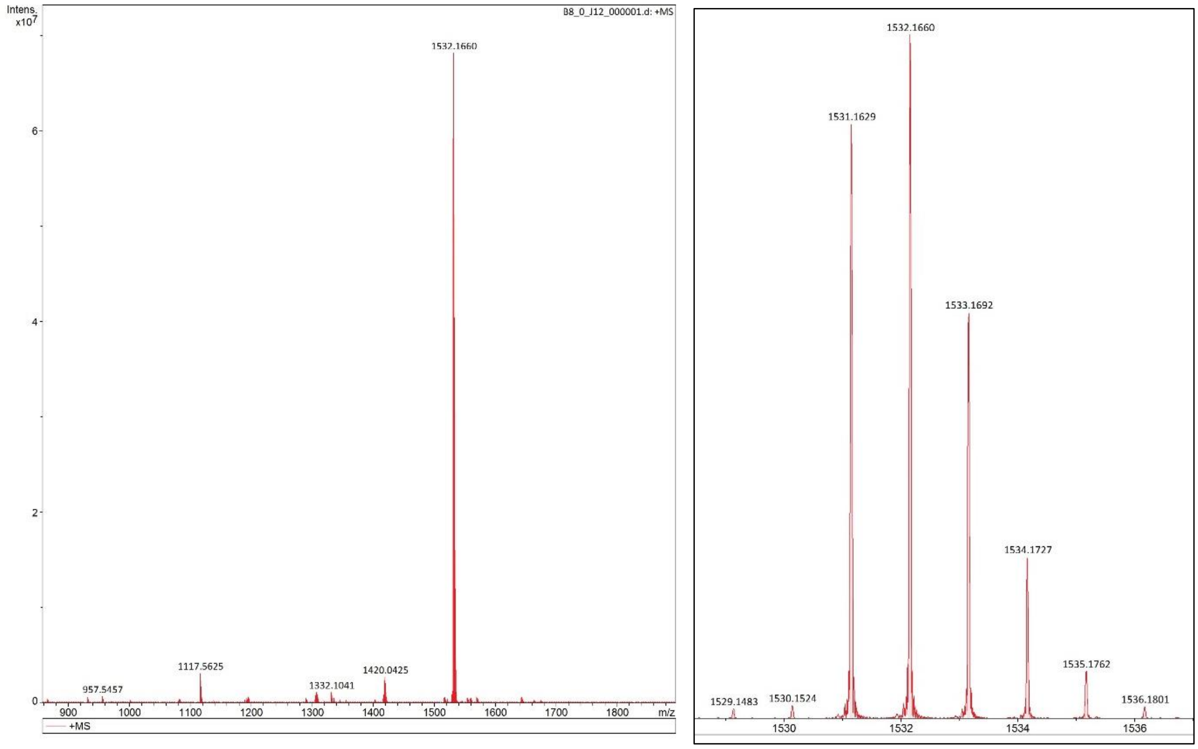

Figure S19. HRMS of **B**8.

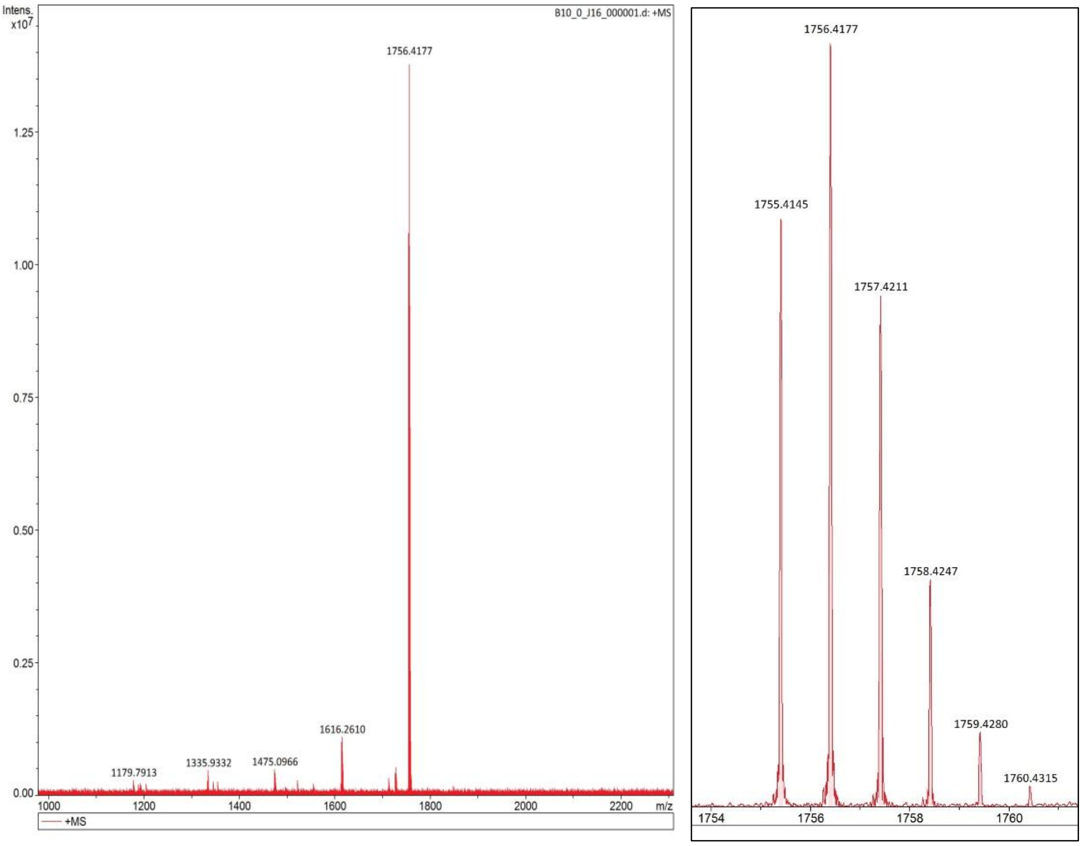

Figure S20. HRMS of **B**10.

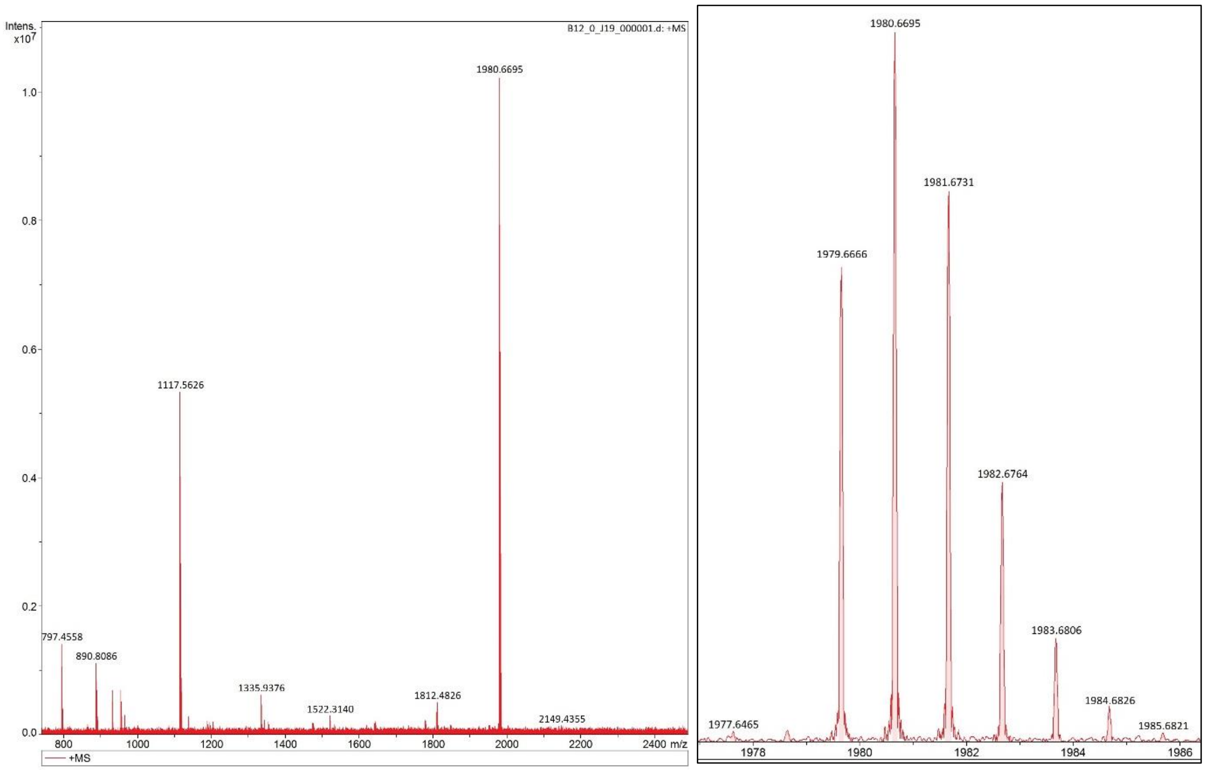

Figure S21. HRMS of **B**12.

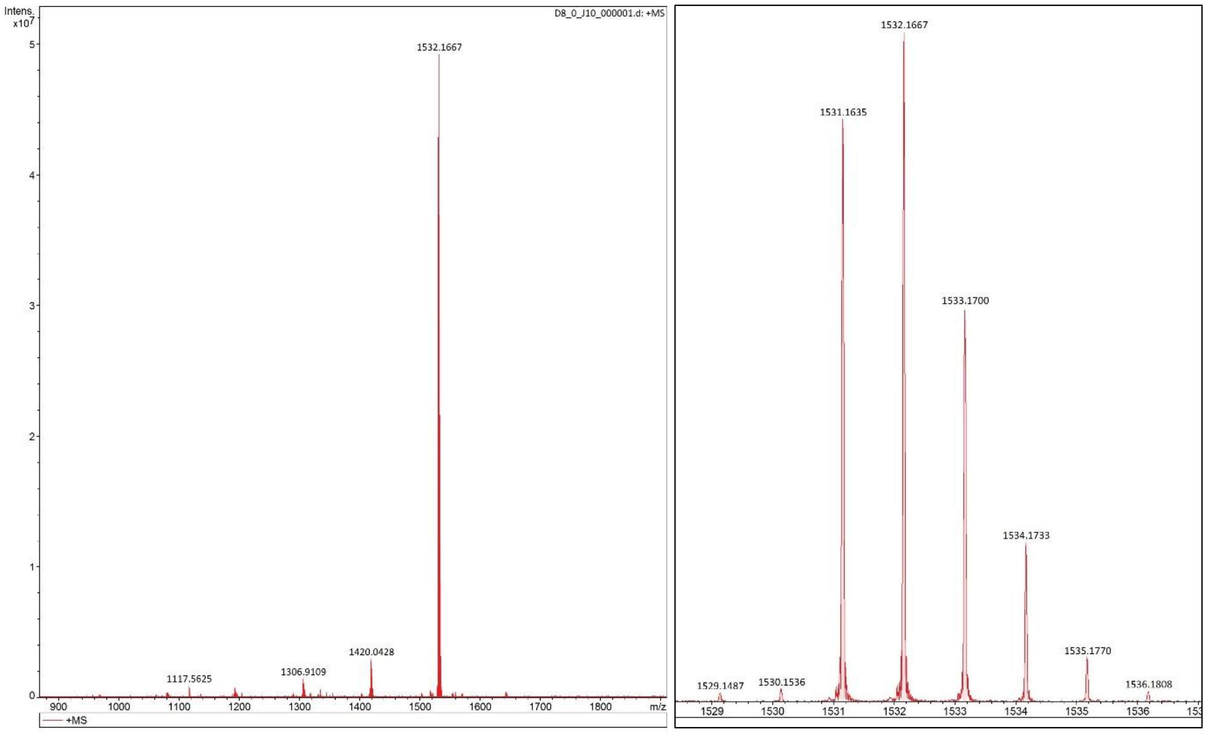

Figure S22. HRMS of **D**8.

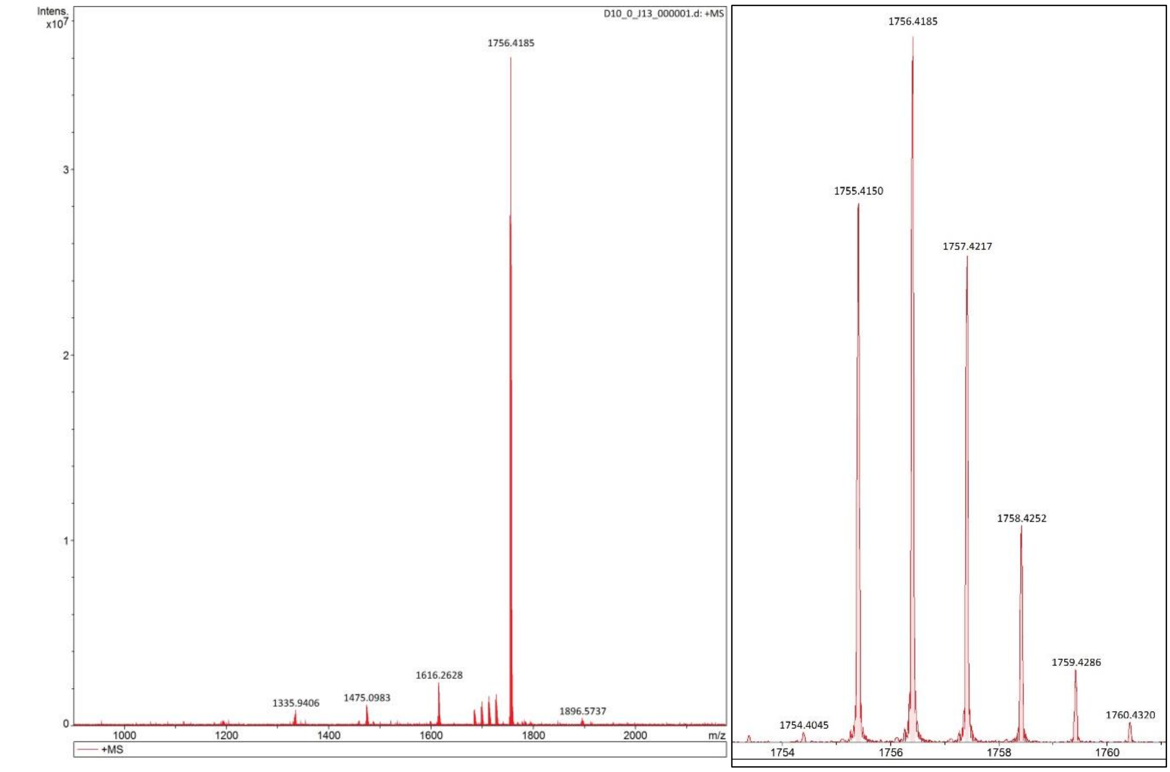

Figure S23. HRMS of **D**10.

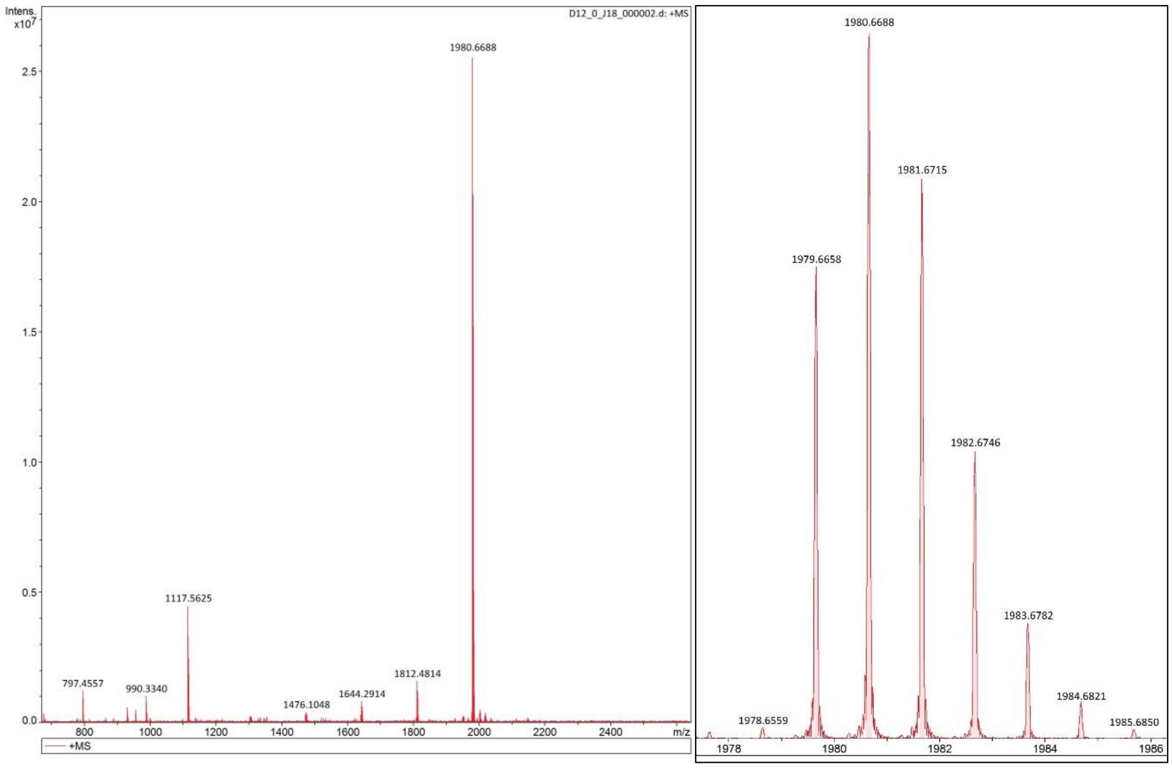

Figure S24. HRMS of **D**12.

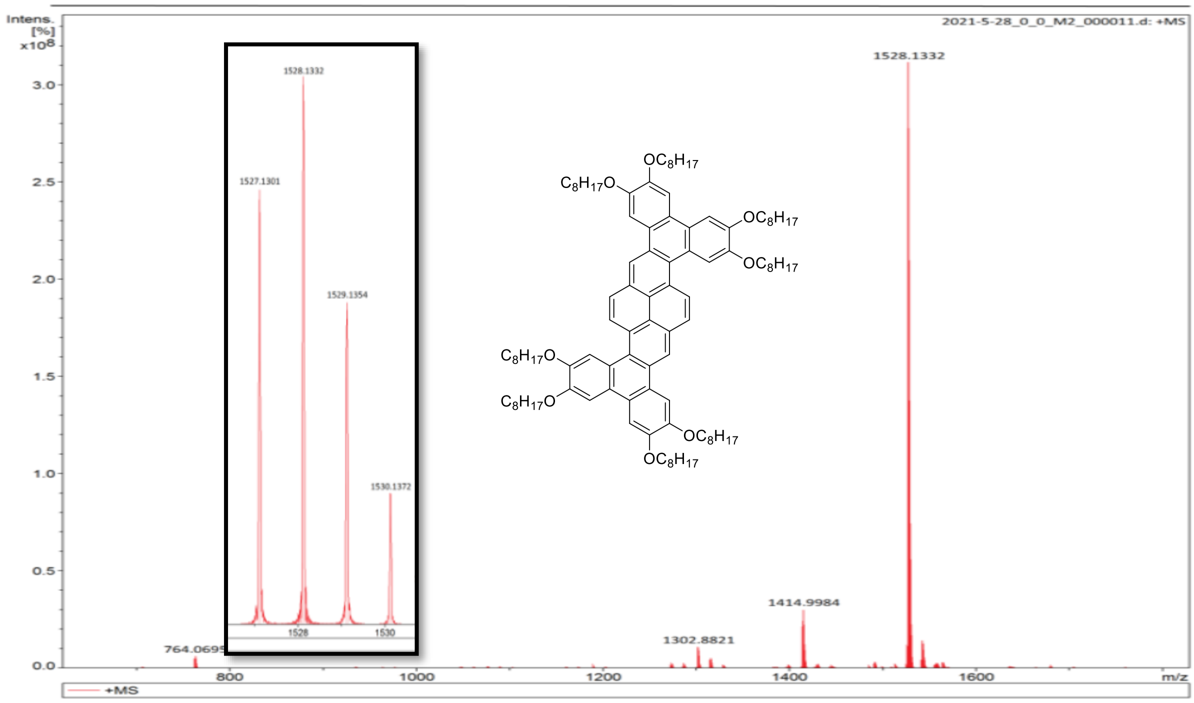

Figure S25. HRMS of **DBP**8.

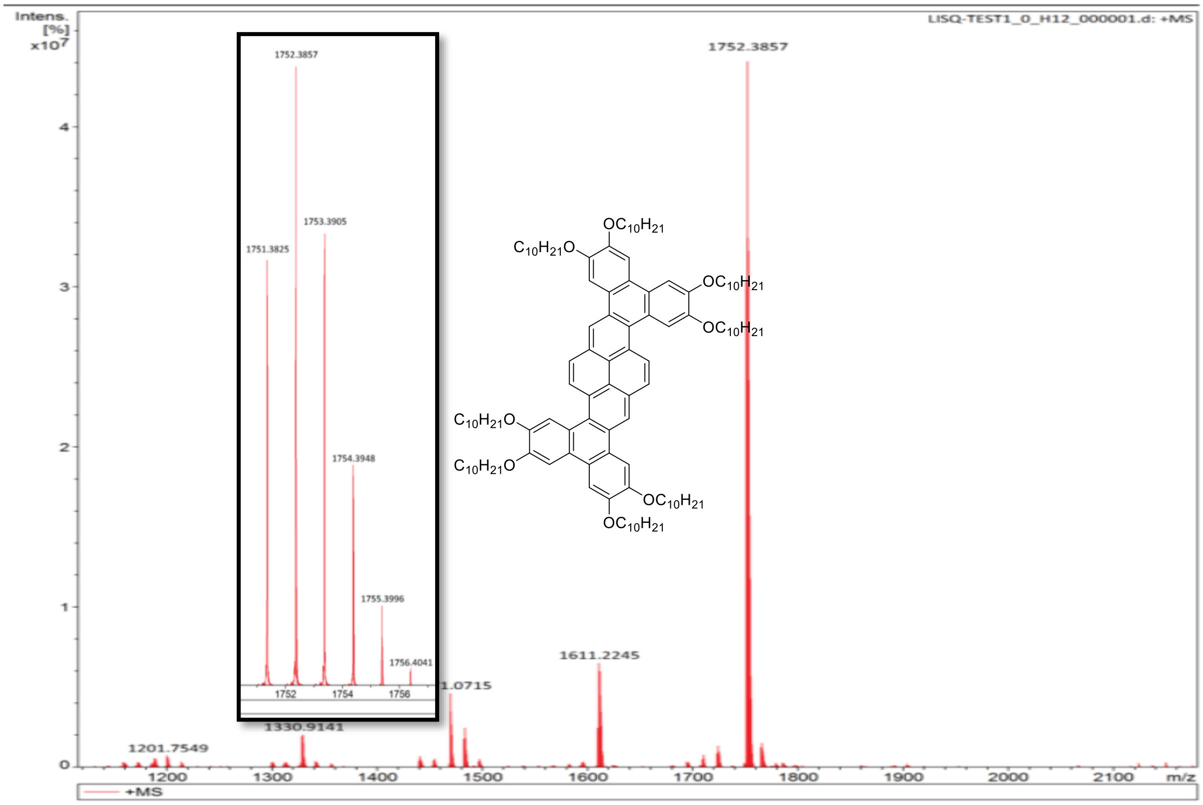

Figure S26. HRMS of **DBP**10.

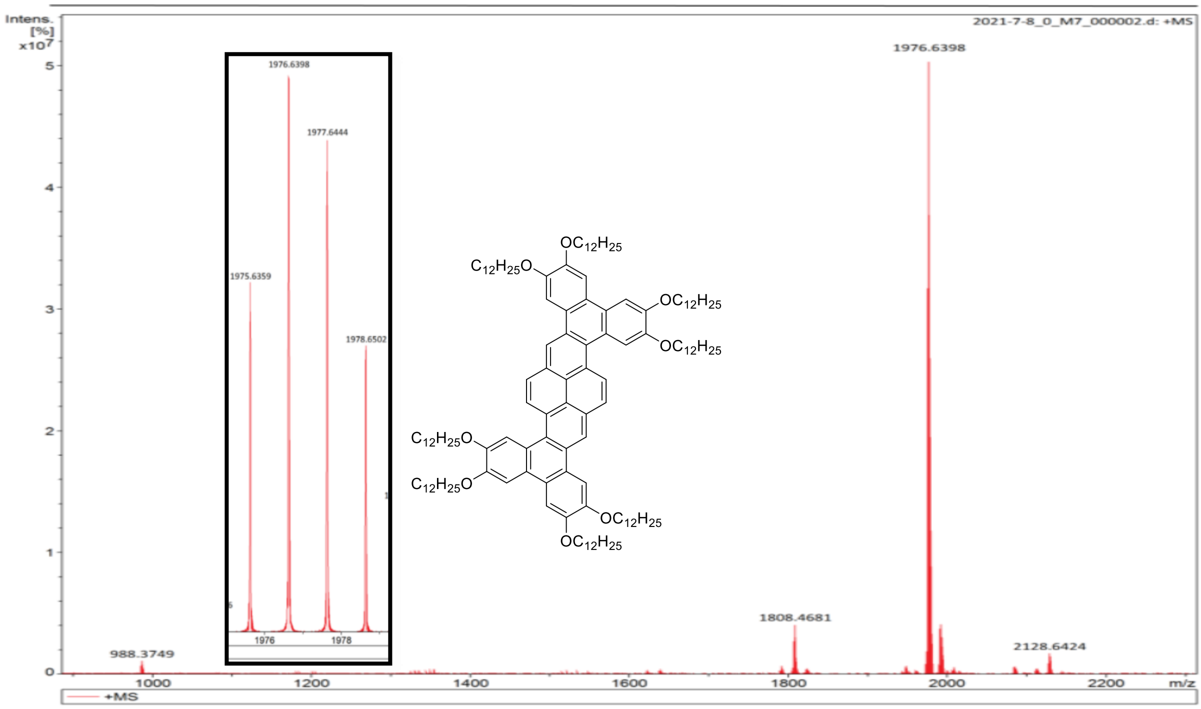

Figure S27. HRMS of **DBP**12.

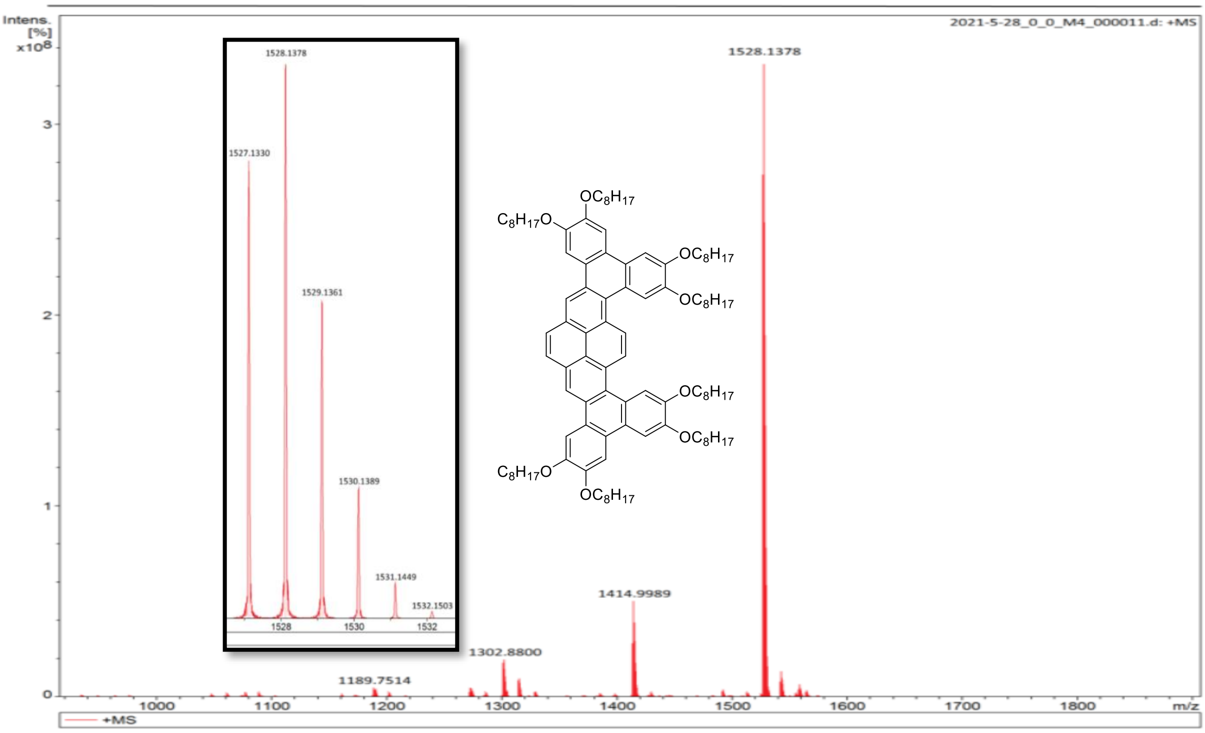

Figure S28. HRMS of **BBP**8.

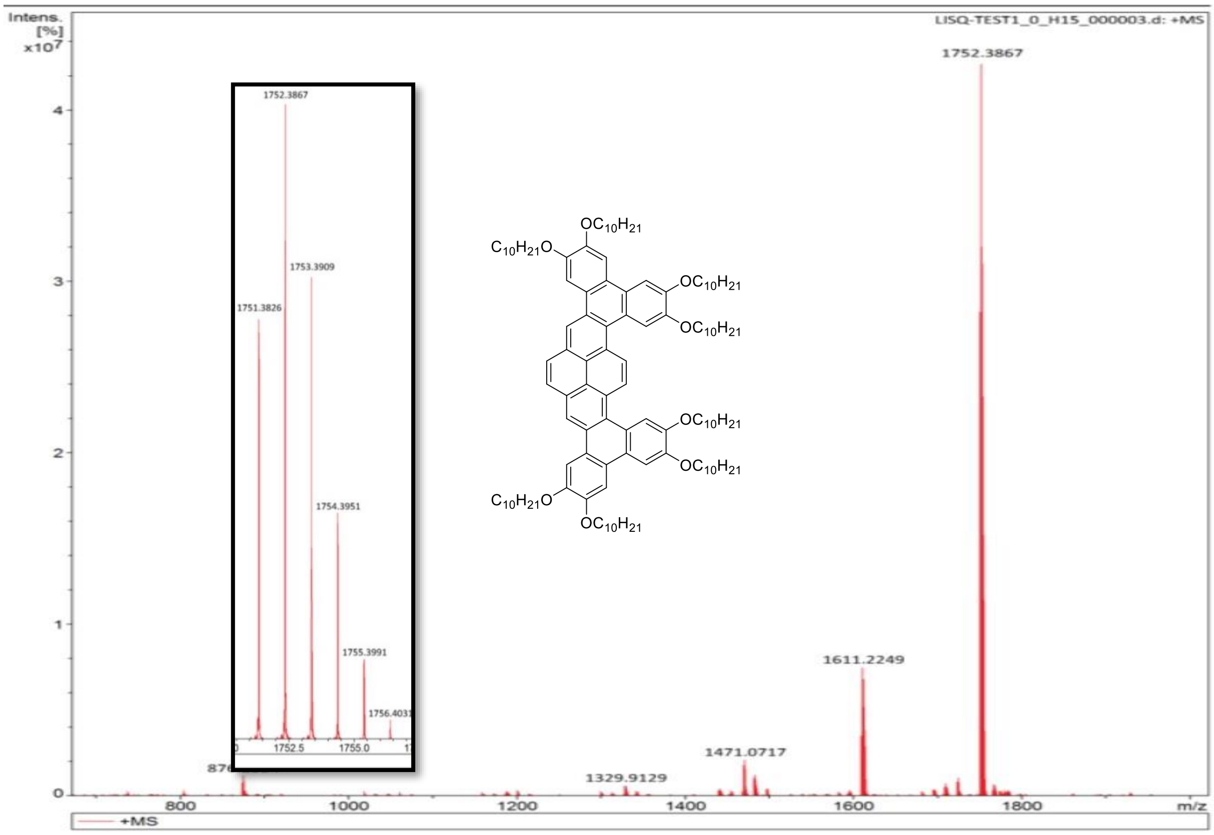

Figure S29. HRMS of **BBP**10.

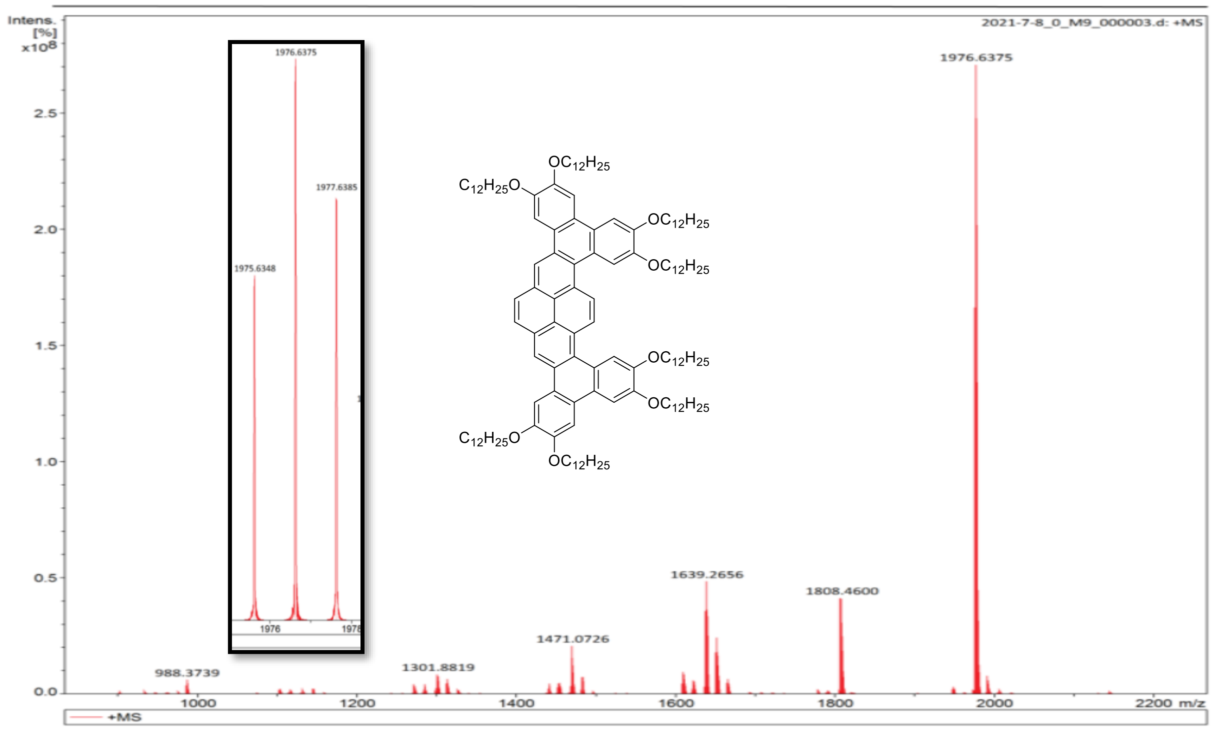

Figure S30. HRMS of **BBP**12.

5. TGA

Table S1. Temperatures of decomposition at 1%, 2% and 5% weight-loss for **BBP***n* and **DBP***n*.

Compd.	T_{dec}/°C(1% loss)	T_{dec}/°C(2% loss)	T_{dec}/°C(5% loss)
**BBP**8	363	377	394
**BBP**10	363	377	393
**BBP**12	363	378	395
**DBP**8	366	380	396
**DBP**10	364	378	394
**DBP**12	362	376	393

6. DSC

Table S2. Mesophases, transition temperatures and enthalpy changes for **DBP**8/10/12 and **BBP**8/10/12 (DSC heating/cooling rate is 10 ºC/min).

Cpds	Mesophases, transition temperature and enthalpy changes	
	2nd heating / ºC (ΔH, kJ·mol^{-1})	1st cooling / ºC (ΔH, kJ·mol^{-1})
**DBP**8	Cr -15 (3.3) Cr' 123 (-6.0) Cr" 130 (43.4) Col_{hex} 185 (2.0) I	I 181 (-1.7) Col_{hex} 104 (-25.9) Cr' 70 (-2.1) Cr
**DBP**10	Cr 12 (6.7) Cr' 97 (-7.5) Cr" 127 (129.5) Col_{hex} 168 (5.3) I	I 167 (-4.2) Col_{hex} 100 (-28.6) Cr" 66 (-2.1) Cr' 8 (-5.0) Cr
**DBP**12	Cr 31 (4.5) Cr' 98 (-7.6) Cr" 119 (127.3) Col_{hex} 153 (3.9) I	I 150 (-3.0) Col_{hex} 99 (-26.6) Cr' 27 (-2.9) Cr
**BBP**8	Cr 60 (2.5) Cr' 67 (-4.1) Cr" 82 (42.1) Col_{rec} 95 (1.5) Col_{hex} 275 (5.8) I	I 272 (-5.9) Col_{hex} 93 (-1.7) Col_{rec} 26 (-51.2) Cr
**BBP**10	Cr 61 (52.3) Col_{hex} 248 (5.1) I	I 246 (-6.1) Col_{hex} 35 (-56.7) Cr
**BBP**12	Cr 66 (92.8) Col_{hex} 235 (6.5) I	I 231 (-9.9) Col_{hex} 45 (-100.8) Cr

Abbreviations: Cr/Cr'/Cr"/Cr'": crystalline phases; Col_{hex}: columnar hexagonal phase; Col_{rec}: columnar rectangular phase; I: isotropic liquid.

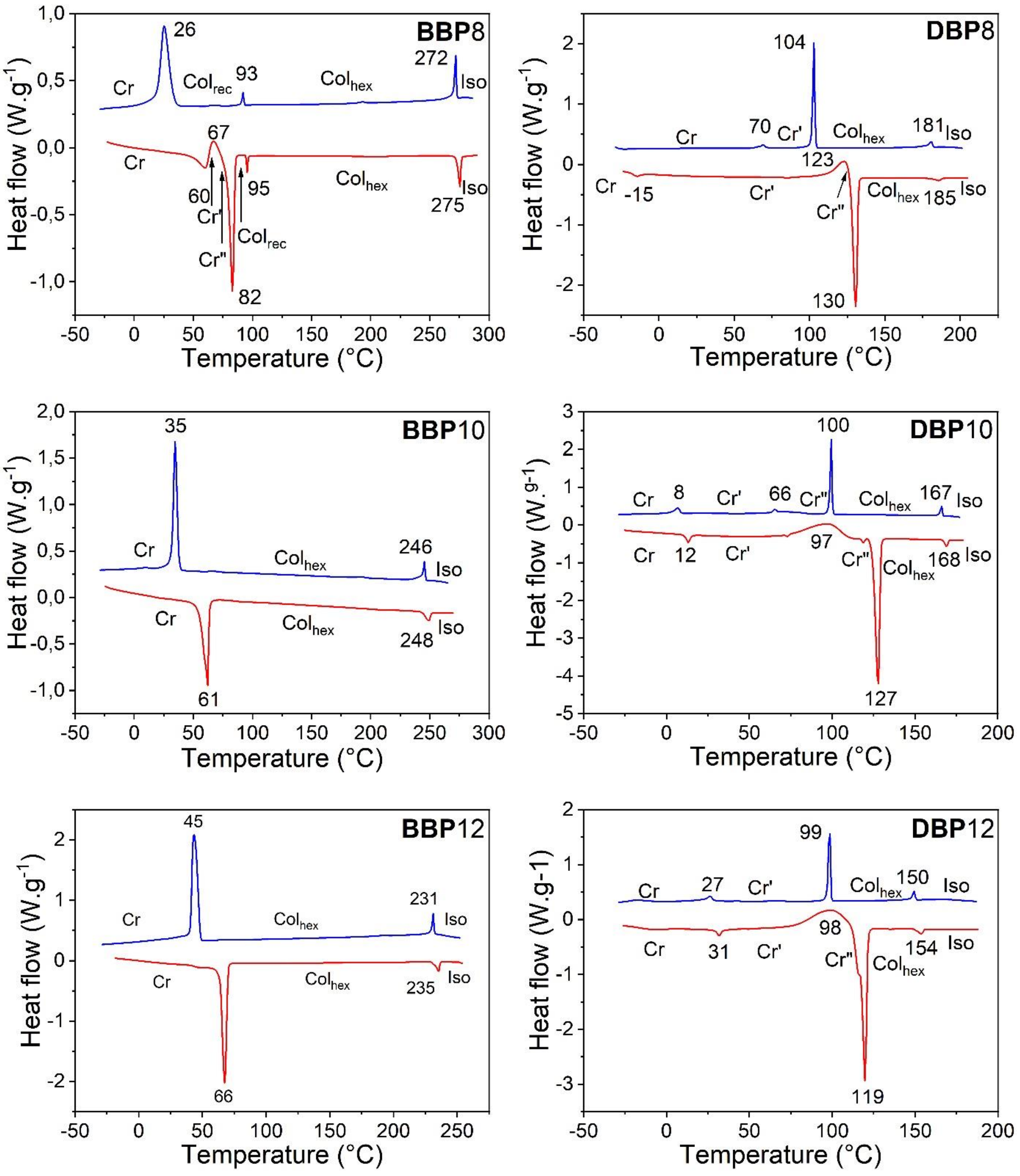

Figure S31. DSC traces of **BBP***n* and **DBP***n* at scanning rate of 10 °C/min (heating red, cooling in blue).

7. POM

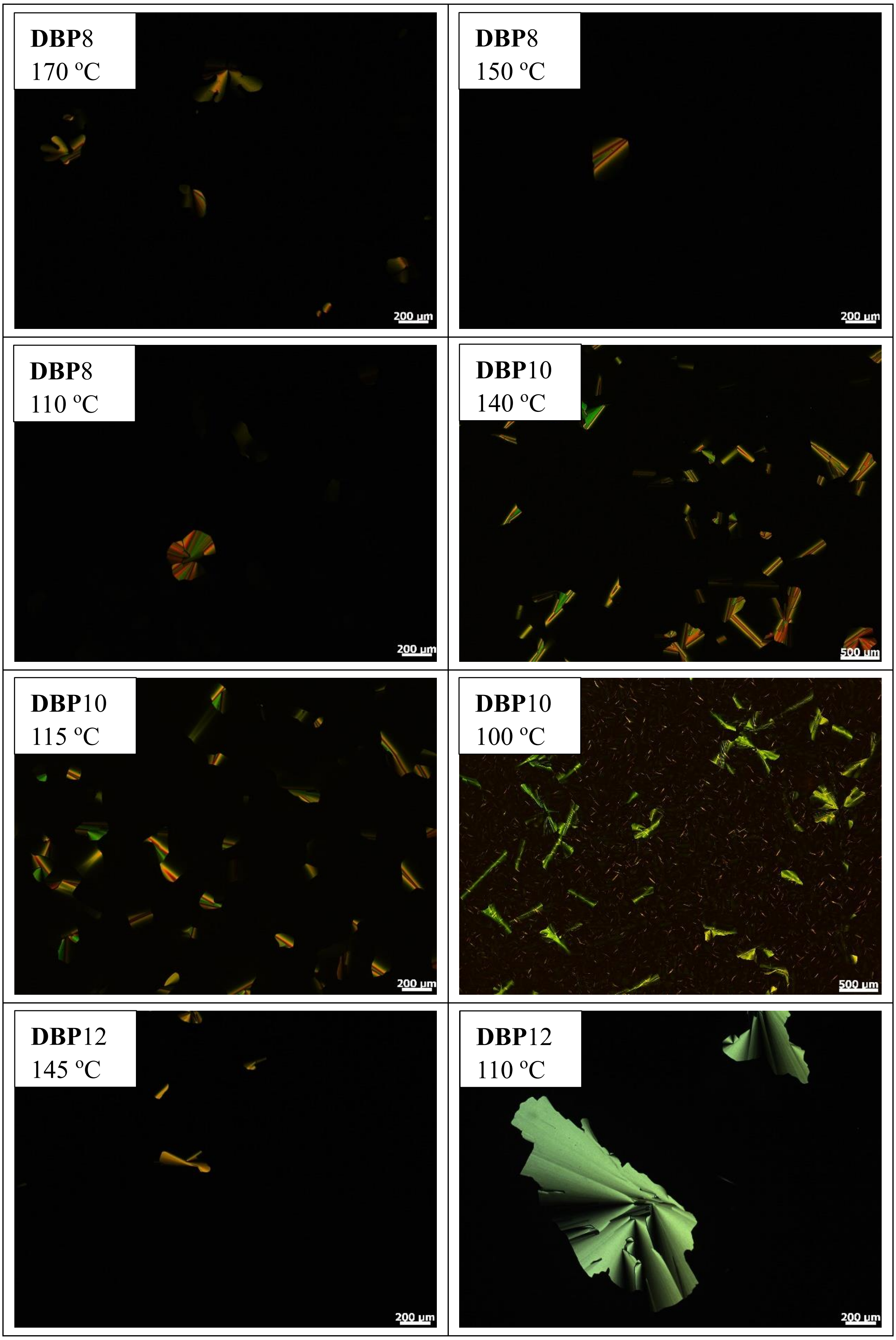
DBP8
170 °C
200 μm
DBP8
150 °C
200 μm
DBP8
110 °C
200 μm
DBP10
140 °C
500 μm
DBP10
115 °C
200 μm
DBP10
100 °C
500 μm
DBP12
145 °C
200 μm
DBP12
110 °C
200 μm

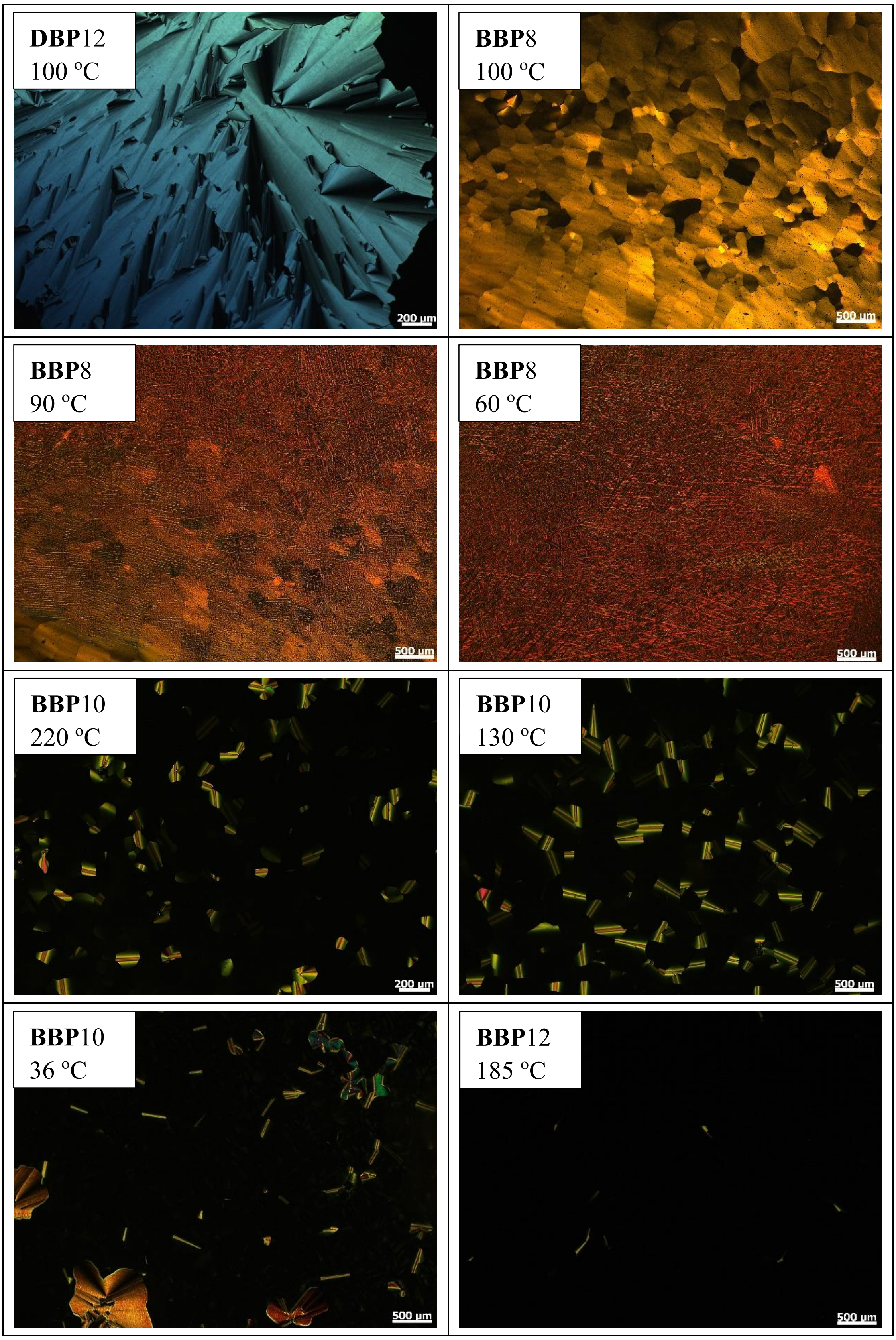
DBP12
100 ºC
200 µm
BBP8
100 ºC
500 µm
BBP8
90 ºC
500 µm
BBP8
60 ºC
500 µm
BBP10
220 ºC
200 µm
BBP10
130 ºC
500 µm
BBP10
36 ºC
500 µm
BBP12
185 ºC
500 µm

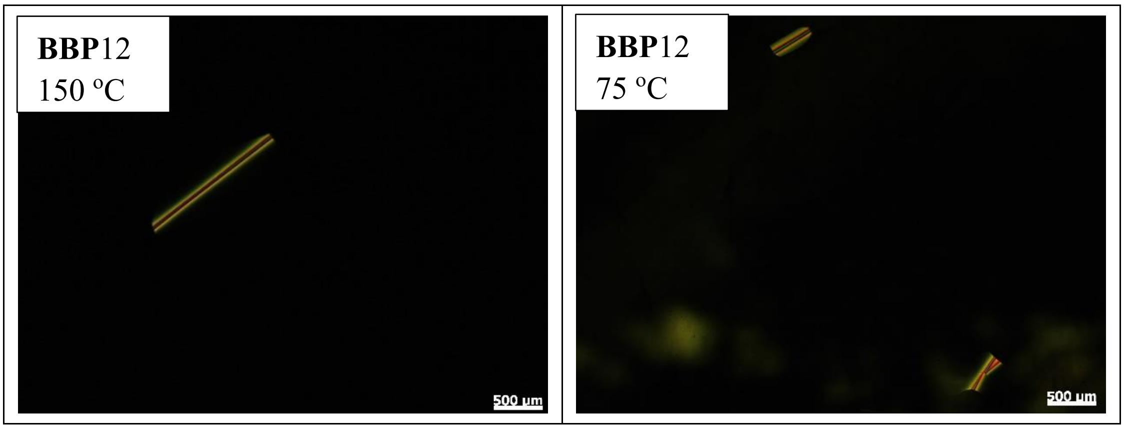

Figure S32. POM textures of **DBP***n* and **BBP***n* on cooling from the isotropic liquid.

8. SWAXS

Table S3. Indexation and geometrical parameters of the columnar mesophases of **DBP***n* and **BBP***n*.

Compd.	2θ $_{exp}$ (°)	d_{exp} (Å)	d_{cal} (Å)	I (%)	*hk*	Lattice parameters
**BBP**8 (87°C)	3.282	26.90	-	S	11	a = 53.80 Å, b = 24.05 Å, A = 1293.89 Å^2
	4.020	21.96	-	VS	20	
	6.566	13.45	13.45	W	40	
	7.373	11.98	12.02	VW	02	
	8.045	10.98	10.98	VW	22	
	8.934	9.89	9.82	VW	51	
	9.776	9.04	8.97	VW	60	
	20.70	4.29	-	S	h_{ch}	
	23.94	3.71	-	VS	h_{π}	
**BBP**8 (120°C)	3.771	23.41	-	VS	10	a = 27.03 Å, A = 632.82 Å^2
	20.53	4.32	-	VW	h_{ch}	
	23.80	3.74	-	VS	h_{π}	
**BBP**8 (240°C)	3.744	23.58	-	VS	10	a = 27.23 Å, A = 641.98 Å^2
	20.42	4.34	-	VW	h_{ch}	
	23.15	3.84	-	VS	h_{π}	
**BBP**10 (80°C)	3.615	24.42	-	VS	10	a = 28.20 Å, A = 688.60 Å^2
	20.54	4.32	-	VW	h_{ch}	
	24.04	3.70	-	VS	h_{π}	
**BBP**10 (160°C)	3.590	24.59	-	VS	10	a = 28.39 Å, A = 698.22 Å^2
	20.19	4.39	-	VW	h_{ch}	
	23.59	3.77	-	VS	h_{π}	
**BBP**10 (230°C)	3.585	24.62	-	VS	10	a = 28.43 Å, A = 700.17 Å^2
	19.69	4.51	-	VW	h_{ch}	
	22.98	3.87	-	VS	h_{π}	
**BBP**12 (75°C)	3.335	26.47	26.48	VS	10	a = 30.56 Å, A = 809.04 Å^2
	5.770	15.30	15.29	VW	11	
	20.11	4.41	-	VS	h_{ch}	
	23.94	3.71	-	VS	h_{π}	
**BBP**12 (160°C)	3.323	26.56	26.56	VS	10	a = 30.67 Å, A = 814.56 Å^2
	5.756	15.34	15.34	VW	11	
	20.29	4.37	-	VS	h_{ch}	
	23.48	3.78	-	VS	h_{π}	
**BBP**12 (200°C)	3.310	26.67	26.67	VS	10	a = 30.79 Å, A = 821.32 Å^2
	5.733	15.40	15.40	VW	11	
	19.57	4.53	-	VS	h_{ch}	
	22.91	3.88	-	VS	h_{π}	

**DBP**8<br>(120°C)	3.857<br>20.72<br>23.98	22.89<br>4.28<br>3.71	-<br>-<br>-	VS<br>VW<br>VS	10<br>h_{ch}<br>h_{π}	a = 26.43 Å<br>A = 604.93 Å^2
**DBP**8<br>(165°C)	3.851<br>20.77<br>23.61	22.92<br>4.27<br>3.76	-<br>-<br>-	VS<br>VW<br>VS	10<br>h_{ch}<br>h_{π}	a = 26.47 Å<br>A = 606.81 Å^2
**DBP**10<br>(110°C)	3.604<br>20.35<br>24.02	24.49<br>4.36<br>3.70	-<br>-<br>-	VS<br>VW<br>VS	10<br>h_{ch}<br>h_{π}	a = 28.28 Å<br>A = 692.81 Å^2
**DBP**10<br>(150°C)	3.570<br>20.17<br>23.89	24.06<br>4.39<br>3.72	-<br>-<br>-	VS<br>VW<br>VS	10<br>h_{ch}<br>h_{π}	a = 28.55 Å<br>A = 706.06 Å^2
**DBP**12<br>(125°C)	3.354<br>5.812<br>20.56<br>23.87	26.31<br>15.19<br>4.32<br>3.72	26.31<br>15.19<br>-<br>-	VS<br>VW<br>VS<br>VS	10<br>11<br>h_{ch}<br>h_{π}	a = 30.38 Å<br>A = 799.30 Å^2
**DBP**12<br>(140°C)	3.327<br>5.754<br>20.64<br>23.61	26.53<br>15.35<br>4.30<br>3.76	26.56<br>15.33<br>-<br>-	VS<br>VW<br>VS<br>VS	10<br>11<br>h_{ch}<br>h_{π}	a = 30.67 Å<br>A = 814.56 Å^2

Annotation: $2\theta_{exp}$: measured diffraction angles corresponding to each peak; d_{exp} and d_{cal}: measured and calculated distance corresponding to each peak; hk: Miller indices of columnar lattice reflection; h_{ch}: average distance between alkyl chains; h_{π}: average distance of π-π stacking of molecules; a, b: columnar lattice parameters; A: lattice area.

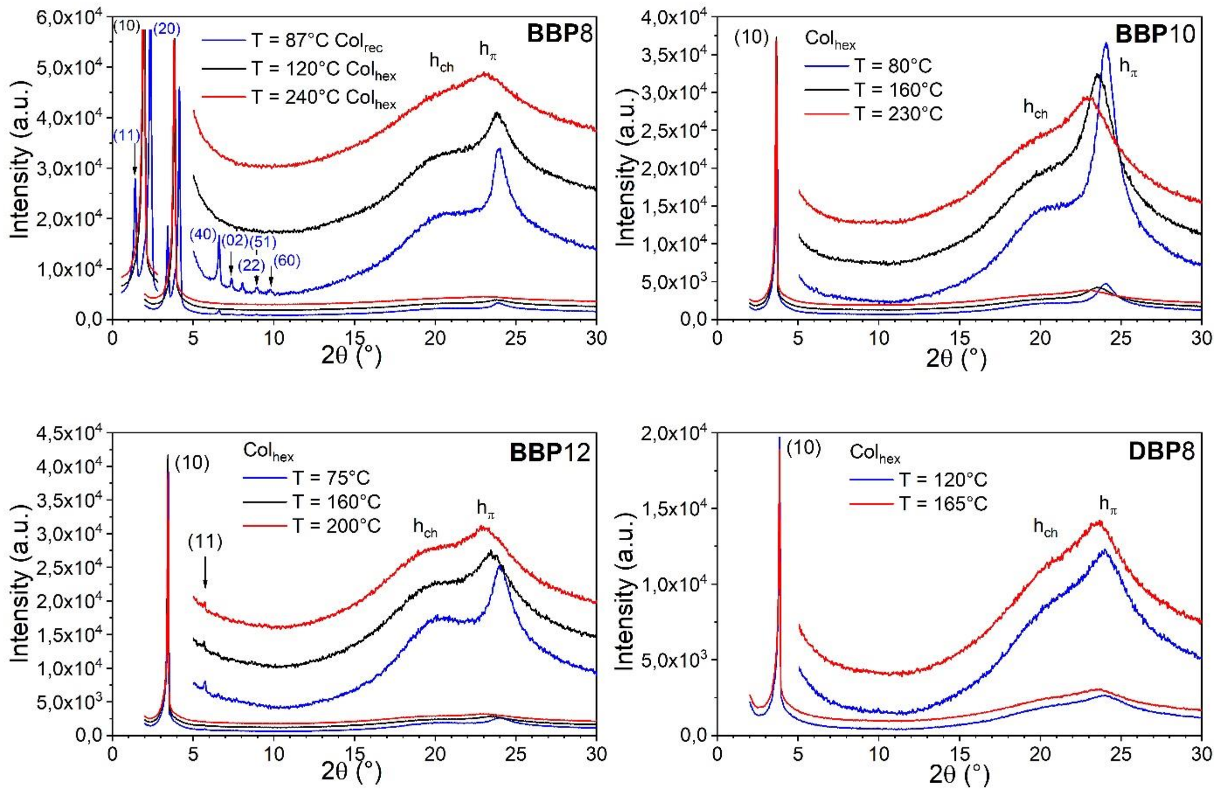

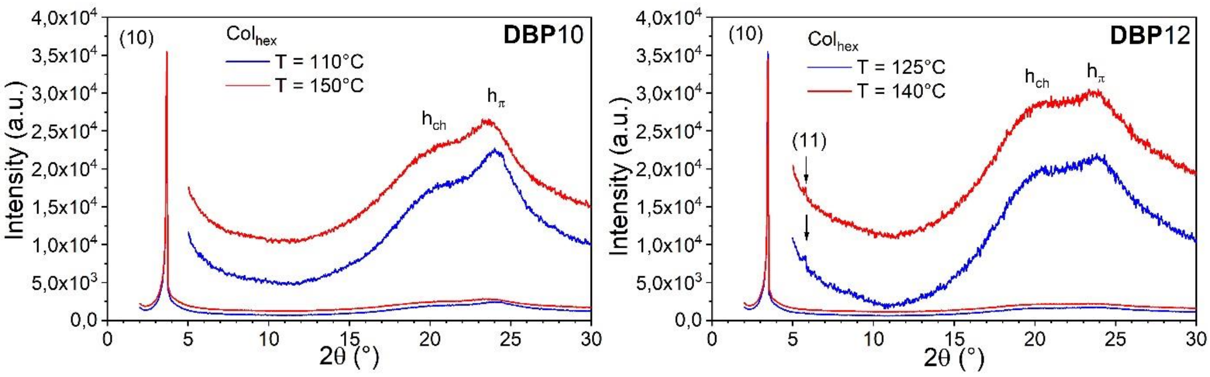

Figure S33. SAXS patterns of the mesophases of compounds **DBP***n* and **BBP***n* (recorded on cooling).

9. DFT Calculation

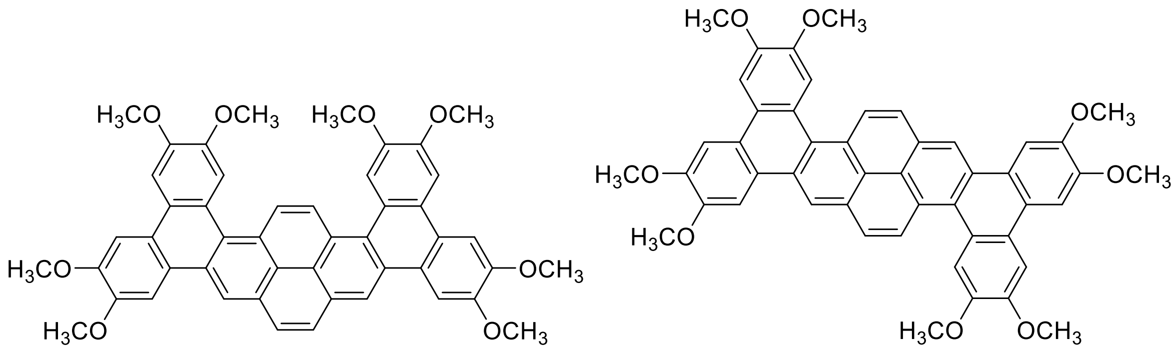

Figure S34. Molecular structures of model compounds **BBP**1 and **DBP**1.

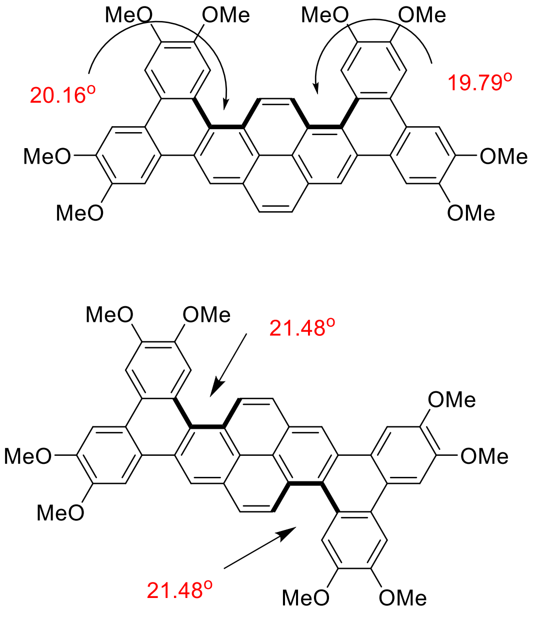

Figure S35. Dihedral angles of compounds **BBP**1 and **DBP**1.

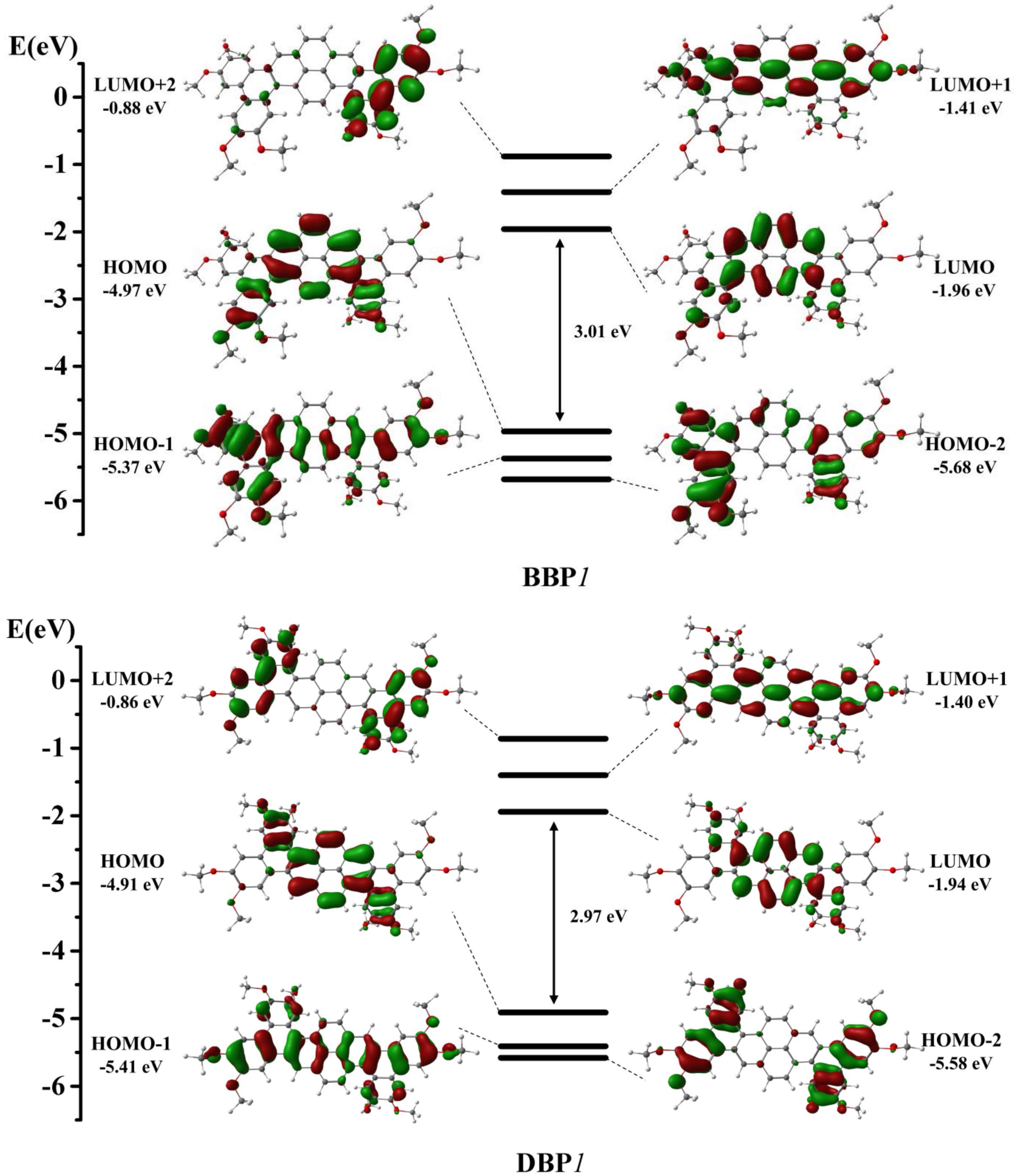

Figure S36. Partial molecular orbital diagram for **BBP**1 and **DBP**1 with some selected isodensity (Isovalues = 0.02) frontier molecular orbital mainly involved in the electronic transitions. All the DFT energy values are given in electronvolts (eV). The arrows are intended to highlight the HOMO-LUMO energy gaps.

Table S4. List of selected molecular orbital energies for **BBP**1 and **DBP**1 and their HOMO-LUMO energy gaps (ΔE).

Compd.	**HOMO-2 (eV)**	**HOMO-1 (eV)**	**HOMO (eV)**	**ΔE (eV)**	**LUMO (eV)**	**LUMO+1 (eV)**	**LUMO+2 (eV)**
**BBP**1	-5.68	-5.37	-4.97	3.01	-1.96	-1.41	-0.88

**DBP**1	-5.58	-5.41	-4.91	2.97	-1.94	-1.40	-0.86

Table S5. Selected calculated excitation energies (ΔE), oscillator strengths (f), main orbital components, and assignment for the **BBP**1 and **DBP**1 in the gas phase.[a]

Compd.	λ_{exc}/nm	ΔE/eV	f	Transitions (Percentage Contribution)
**BBP**1	455.2	2.72	0.6581	H→L(+69%)
	441.0	2.81	0.0039	H-1→L(+55%), H→L+1(+43%)
	390.8	3.17	0.0196	H-2→L(+46%), H-1→L(+45%)
	379.2	3.27	0.0087	H-3→L(+67%)
	367.7	3.37	0.1645	H-2→L(+50%), H→L+1(+39%)
	349.2	3.55	0.9634	H-1→L+1(+63%), H→L+3(+15%), H→L+2(+14%)
	343.3	3.61	0.0377	H→L+2(+63%)
	337.5	3.67	0.0757	H-4→L(+46%), H-2→L+1(+29%), H-1→L+3(+11%)
	336.7	3.68	0.2251	H→L+3(+62%), H→L+2(+13%)
	325.2	3.81	0.2052	H-2→L+1(+50%), H→L+4(+26%), H-1→L+3(+11%)
	325.0	3.82	0.0249	H→L+4(+52%), H-4→L+0(+28%), H-3→L+1(+19%)
	323.1	3.84	0.0063	H-3→L+1(+54%), H-1→L+3(+17%), H→L+2(+16%)
	319.1	3.89	0.0035	H→L+5(+50%), H→L+6(+22%), H-5→L(+15%)
	308.0	4.03	0.0299	H-5→L(+48%), H-1→L+3(+40%), H-1→L+2(+18%)
	304.6	4.07	0.0940	H→L+6(+52%), H-1→L+2(+29%), H-2→L+1(+24%)
	297.7	4.16	0.1027	H-1→L+2(+39%), H→L+5(+18%), H-2→L+1(+16%)
	295.9	4.19	0.1444	H-1→L+4(+36%), H→L+4(+19%), H-4→L+1(+15%)
	293.5	4.22	0.0490	H-1→L+4(+46%), H-1→L+5(+27%), H-5→L(+19%)
	289.2	4.29	0.0696	H-4→L+1(+40%), H-5→L(+14%)
	287.6	4.31	0.0044	H-9→L(+42%), H-8→L(+32%), H-7→L(+13%)
	285.7	4.34	0.0019	H-6→L(+49%), H-9→L(+30%), H-8→L(+19%)
	284.2	4.36	0.0660	H-2→L+2(+41%), H-4→L+1(+30%), H-2→L+3(+22%)
	280.6	4.42	0.1374	H-2→L+2(+37%), H-7→L(+36%)
	279.2	4.44	0.1009	H-7→L(+40%), H-1→L+5(+21%), H-1→L+6(+17%)
	278.2	4.46	0.1852	H-2→L+3(+32%), H-7→L(+30%), H-3→L+2(+27%)
	275.1	4.51	0.0273	H-3→L+3(+39%), H-2→L+4(+30%), H-3→L+2(+23%)
	274.6	4.52	0.1989	H-8→L(+36%), H-2→L+3(+18%), H-2→L+4(+13%)
	271.8	4.56	0.0927	H-1→L+6(+50%), H-3→L+2(+22%), H-4→L+1(+18%)
	271.4	4.57	0.0107	H-1→L+6(+28%), H-3→L+3(+25%), H-2→L+2(+21%)
	269.2	4.61	0.1048	H-5→L+1(+34%), H-2→L+3(+25%), H-3→L+3(+21%)
	267.5	4.63	0.0378	H-2→L+4(+37%), H→L+7(+29%), H-5→L+1(+21%)
	265.3	4.67	0.1153	H-3→L+4(+43%), H-2→L+3(+21%), H-1→L+6(+17%)
	264.6	4.69	0.2382	H-2→L+5(+44%), H-3→L+4(+28%), H-2→L+3(+19%)
	263.0	4.71	0.2071	H-3→L+5(+51%), H-5→L+1(+28%), H-2→L+2(+14%)
	262.2	4.73	0.0699	H-10→L(+57%), H-2→L+5(+19%), H-6→L+1(+19%)
	258.7	4.79	0.0986	H→L+7(+50%), H-4→L+2(+16%), H-3→L+3(+14%)
	254.1	4.88	0.0261	H-4→L+2(+44%), H-6→L+1(+35%), H-1→L+5(+12%)

	252.9	4.90	0.0762	H-4→L+3(+39%), H-4→L+2(+22%)
	251.9	4.92	0.0381	H-2→L+6(+40%), H→L+8(+29%), H-10→L(+14%)
	250.8	4.94	0.0582	H-4→L+3(+40%), H-2→L+6(+29%), H-6→L+1(+27%)
	250.1	4.96	0.0107	H→L+8(+44%), H-11→L(+31%), H-6→L+1(+11%)
	249.2	4.97	0.0606	H-3→L+6(+49%), H-4→L+2(+17%), H-8→L+1(+11%)
	247.7	5.01	0.0117	H-9→L+1(+48%), H-7→L+1(+21%), H-8→L+1(+18%)
	246.6	5.03	0.0260	H-8→L+1(+34%), H-3→L+6(+13%), H-6→L+2(+13%)
	245.0	5.06	0.0227	H-8→L+1(+39%), H-4→L+4(+28%), H-7→L+1(+24%)
	243.8	5.09	0.0192	H-4→L+4(+50%), H-5→L+3(+16%), H→L+8(+14%)
	243.1	5.10	0.0025	H-11→L(+35%), H-4→L+5(+12%)
	242.1	5.12	0.0453	H-1→L+7(+43%), H-11→L(+32%), H-5→L+3(+18%)
	241.1	5.14	0.0340	H-5→L+2(+45%), H-4→L+5(+21%), H-8→L+1(+17%)
	238.3	5.20	0.0549	H-4→L+5(+53%), H-13→L+0(+27%), H-5→L+3(+11%)
**DBP**1	462.1	2.68	0.5798	H→L(+69%), H-1→L+1(+12%)
	439.5	2.82	0.0014	H-1→L(+50%)
	393.2	3.15	0.0004	H-2→L(+70%)
	378.2	3.28	0.6872	H-1→L(+48%), H→L+1(+47%), H-3→L(+16%)
	357.1	3.47	0.0000	H-4→L(+54%), H→L+4(+31%)
	351.9	3.52	0.2981	H-3→L(+52%), H-1→L+1(+27%)
	346.5	3.58	0.0083	H→L+2(+65%)
	341.8	3.63	0.4925	H-1→L+1(+50%)
	336.9	3.68	0.4815	H→L+3(+56%), H-1→L+1(+37%), H-3→L(+18%)
	332.9	3.72	0.0004	H-2→L+1(+55%), H→L+4(+24%), H→L+2(+19%)
	328.6	3.77	0.0114	H→L+4(+51%), H-1→L+2(+22%), H→L+6(+15%)
	316.4	3.92	0.0029	H→L+5(+62%), H-1→L+2(+11%)
	314.0	3.95	0.1723	H-3→L+1(+51%), H-2→L+4(+16%), H-4→L+2(+15%)
	305.9	4.05	0.0007	H→L+6(+59%), H-4→L+1(+21%)
	301.8	4.11	0.0016	H-5→L(+45%), H→L+6(+23%), H-2→L+3(+13%)
	299.5	4.14	0.0163	H-4→L+1(+47%), H-1→L+2(+32%), H-5→L(+30%)
	295.6	4.19	0.1113	H-1→L+3(+46%), H-3→L+1(+36%)
	293.5	4.22	0.0288	H-1→L+4(+38%), H-5→L(+22%), H-2→L+3(+18%)
	290.4	4.27	0.0154	H-8→L(+37%)
	287.3	4.32	0.0281	H-6→L(+42%), H-3→L+1(+21%), H-7→L(+18%)
	286.7	4.33	0.0097	H-8→L(+40%), H-1→L+4(+26%), H-1→L+5(+16%)
	281.9	4.40	0.5011	H-2→L+2(+51%), H-6→L(+26%), H-3→L+1(+17%)
	280.3	4.42	0.0030	H-1→L+5(+38%), H-2→L+3(+29%)
	278.6	4.45	0.0310	H-2→L+4(+38%), H-1→L+3(+24%), H-6→L(+22%)
	277.4	4.47	0.0761	H-9→L(+38%), H-2→L+3(+36%), H-8→L(+22%)
	276.8	4.48	0.1209	H-7→L(+48%), H-2→L+4(+33%), H-2→L+2(+21%)
	274.4	4.52	0.0532	H-9→L(+42%), H-8→L(+31%), H-1→L+5(+24%)
	269.1	4.61	0.0022	H-2→L+5(+62%), H→L+7(+17%)
	266.2	4.66	0.0001	H-1→L+6(+43%), H-8→L+1(+21%)
	265.9	4.66	0.0025	H-3→L+2(+36%)
	265.5	4.67	0.0805	H→L+7(+41%), H-4→L+2(+38%), H-10→L(+23%)

	264.0	4.70	0.0659	H-3→L+3(+44%), H-4→L+2(+12%)
	262.0	4.73	0.0561	H-3→L+2(+46%), H-1→L+6(+32%), H-4→L+3(+22%)
	259.6	4.78	0.0848	H-3→L+3(+38%), H-10→L(+34%), H-6→L+1(+34%)
	257.7	4.81	0.0246	H-6→L+1(+34%), H-4→L+2(+34%), H-4→L+4(+28%)
	257.1	4.82	0.0883	H-5→L+1(+53%), H-3→L+2(+32%), H-1→L+5(+18%)
	255.2	4.86	0.2925	H-10→L(+37%), H-4→L+5(+17%), H-4→L+2(+14%)
	254.8	4.87	0.0143	H-3→L+4(+48%), H-3→L+5(+11%)
	253.1	4.90	0.3586	H-4→L+4(+47%), H-3→L+3(+16%), H-4→L+5(+15%)
	251.1	4.94	0.0466	H-2→L+6(+59%), H-7→L+1(+18%), H-4→L+2(+15%)
	250.4	4.95	0.0382	H-3→L+5(+51%), H-8→L+1(+23%), H→L+9(+15%)
	249.2	4.98	0.0068	H-8→L+1(+49%), H-11→L(+30%), H-3→L+4(+22%)
	249.0	4.98	0.0608	H-7→L+1(+51%)
	247.5	5.01	0.3177	H→L+8(+50%), H-10→L(+29%), H-4→L+4(+18%)
	246.3	5.03	0.0017	H-9→L+1(+37%), H-8→L+1(+27%), H→L+9(+27%)
	246.0	5.04	0.0004	H-9→L+1(+50%), H-11→L(+28%), H-3→L+5(+20%)
	244.8	5.06	0.0725	H-4→L+5(+47%), H-7→L+1(+33%), H-6→L+1(+14%)
	239.5	5.18	0.0139	H-1→L+7(+55%), H-5→L+2(+28%), H-4→L+6(+10%)
	238.5	5.20	0.0003	H-12→L(+58%), H-2→L+7(+12%)
	238.1	5.21	0.0006	H-12→L(+35%), H→L+9(+31%), H-11→L(+27%)

[a]H = HOMO, L = LUMO, H-n = HOMO-n and L+n = LUMO+n.
